# From information quality to episodic discontinuation intention: cognitive and affective processes in social media browsing

**DOI:** 10.3389/fpsyg.2026.1858297

**Published:** 2026-07-02

**Authors:** Lei Tang, Chaohao Wu, Lina Ma, Lufang Wang, Jiewen Han, Xinxin Jiang

**Affiliations:** Yunnan University of Finance and Economics, Kunming, China

**Keywords:** cognitive load, emotional fatigue, episodic discontinuation, information redundancy, information veracity, negative use evaluation

## Abstract

As social media browsing becomes increasingly episodic, users may develop intentions to interrupt browsing without permanently abandoning a platform. Although prior research has linked information overload to fatigue and discontinuance, less is known about how information-quality cues shape users’ immediate browsing experience and short-term discontinuation intention. This study examines the effects of information veracity and redundancy on cognitive load, negative use evaluation, emotional fatigue, and episodic discontinuation intention. A 2 × 2 within-subject experiment manipulated information veracity and redundancy in a simulated social media browsing context. Participants viewed travel-related social media posts and completed self-report measures after exposure, and linear mixed-effects models were used to account for repeated observations nested within participants. Low-veracity and high-redundancy conditions were associated with higher cognitive load and more negative use evaluation. Emotional fatigue positively predicted episodic discontinuation intention. The findings clarify how information-quality cues are linked to users’ short-term discontinuation intention during browsing episodes.

## 1 Introduction

In everyday social media use, engagement is rarely as continuous as it seems. Users may experience momentary intentions to pause, stop reading, or discontinue browsing before a browsing session is fully completed, even though they may return later. These self-reported interruption-related intentions do not necessarily reflect a permanent intention to discontinue platform use. More often, they represent temporary intention states that emerge within ongoing browsing episodes. Understanding why users develop intentions to interrupt browsing has therefore become an important issue in explaining contemporary social media behavior. Such short-term interruption matters because it may signal a deterioration of users’ immediate browsing experience before permanent discontinuance occurs. For platforms, such short-term discontinuation intention may also indicate declining attention, reduced content engagement, and weaker continuity of information exposure.

Existing studies on non-continuous social media use have generally developed along two lines. One emphasizes external pressures, showing that information overload, social overload, functional overload, privacy concerns, and technological risks can increase discontinuation intentions or behaviors (Bright et al., 2015; Li et al., 2025; Liu et al., 2021; Pentina and Tarafdar, 2014; Wang et al., 2021). The other adopts an affective perspective, treating fatigue, anxiety, or emotional exhaustion as central explanations for temporary interruption or withdrawal (Bright et al., 2015; Zhang et al., 2020). These studies have offered valuable insights, but they remain largely focused on outcomes. Fatigue is often treated as a relatively unitary mediator linking stressors to discontinuation. By contrast, much less attention has been paid to how users’ ongoing browsing experiences are cognitively interpreted and gradually develop into emotional fatigue. This limitation becomes especially evident in episodic discontinuation, where users report fluctuating intentions to continue or temporarily discontinue browsing rather than indicating permanent engagement or exit. In such situations, the psychological progression from cognitive burden to evaluative judgment and then to emotional fatigue remains conceptual and is captured via self-reported episodic discontinuation intention.

This gap is important because discontinuance intention may begin to form before users experience stable fatigue or make a clear decision to withdraw. It is therefore necessary to examine the intermediate cognitive and evaluative processes through which information-quality cues become emotionally consequential during browsing.

To address this gap, the present study shifts attention to users’ momentary experiences during ongoing social media browsing. We focus on two information-related cues that are especially salient in browsing contexts, namely information veracity and information redundancy, and examine how they shape cognitive load, negative use evaluation, and emotional fatigue. Rather than assuming that episodic discontinuation arises directly from information exposure, we argue that it takes shape through a staged psychological process. Information cues first increase cognitive burden. This burden is then interpreted through negative use evaluation, and only afterward does it gradually develop into emotional fatigue. We further examine whether emotional fatigue predicts episodic discontinuation intention in short-term tasks, thereby clarifying when subjective experience begins to take on behavioral relevance.

## 2 Literature review

### 2.1 Information environment complexity and user discontinuance

Research on non-continuous social media use has largely approached the issue from the perspective of information environment complexity. Information overload, social overload, and functional overload have been consistently identified as external antecedents of user fatigue and discontinuance (Bright et al., 2015; Eppler, 2004; Maier et al., 2015). While this work has clarified important environmental triggers, it has primarily focused on associations between external conditions and behavioral outcomes, with less attention to how these conditions are internally processed and transformed into psychological experiences during browsing.

More recent research suggests that users’ responses are shaped not only by the amount of information they encounter, but also by the quality and organization of that information. Eye-tracking research shows that structurally complex or unclear information disperses attention and increases processing costs (Rayner, 2009; Pelău et al., 2020). Similarly, studies in social media contexts demonstrate that lower credibility and higher redundancy are associated with avoidance and discontinuance tendencies (Pan et al., 2023).

Although social interaction demands and platform complexity also contribute to usage burden (Bright et al., 2015; Maier et al., 2015), such factors represent relatively stable contextual conditions. In the present study, these contextual influences were held constant. This focus allows us to move from identifying external antecedents of discontinuance toward explaining the internal psychological process through which information-quality differences shape user experience.

### 2.2 Information quality and cognitive load

Information quality is widely conceptualized as a multidimensional construct whose relevance varies across tasks and contexts (Wang and Strong, 1996). In the present study, we focus specifically on two dimensions of information quality: information veracity and information redundancy. These two dimensions are particularly relevant to fragmented social media browsing, where users encounter posts from multiple sources, with varying levels of reliability, repetition, and informational value.

Continuous browsing requires users to rapidly filter and interpret large volumes of content under limited cognitive resources. Under such conditions, users often rely on local cues to judge whether the information is reliable and worth continued attention. Low veracity increases the cognitive effort required to judge accuracy and deal with inconsistent claims. Redundancy, in contrast, consumes attentional resources through repetitive content without adding informational value (Eppler, 2004). Both mechanisms intensify processing burden during sustained browsing.

Over time, repeated exposure to uncertain and redundant content may produce cumulative depletion of cognitive resources. Prior research similarly shows that insufficient credibility and disorganized information structures elevate cognitive load and reduce perceived control (Liu et al., 2021). In the present study, cognitive load is therefore conceptualized as primarily driven by content-induced processing demands, rather than by broader platform or social factors. Differences in information quality are expected to manifest first as variations in cognitive processing burden, forming the antecedent basis for subsequent evaluative and emotional responses.

### 2.3 Negative use evaluation as evaluative interpretation

Cognitive Appraisal Theory suggests that emotional responses are shaped by how individuals interpret the meaning of a situation in relation to their goals and coping resources (Lazarus, 1991; Lazarus and Folkman, 1984). In browsing contexts, users do not respond only to the amount of information they process. They also evaluate whether continued attention remains useful, manageable, and worthwhile.

Within the CAT framework, individuals first engage in primary appraisal, during which they assess the significance of a situation by considering the relationship between an event and their personal goals. This appraisal centers on goal relevance and goal congruence, leading individuals to classify events as irrelevant, benign-positive, or stressful. Notably, stressful events are not homogeneous but can be further differentiated into harm, threat, and challenge. Different appraisal outcomes are associated with distinct emotional experiences and coping pathways (Lazarus, 1991; Lazarus and Folkman, 1984).

In information environments characterized by uncertainty in quality and veracity, sustained information processing and experiences of cognitive load are likely to prompt users to reassess their current usage context. As processing costs accumulate, users may gradually form holistic judgments that the current information no longer supports their usage goals or that continued attentional investment is no longer worthwhile. From the perspective of cognitive appraisal theory, such judgments do not reflect a generic evaluation of stress. Instead, they are more closely related to threat-oriented appraisal within primary appraisal, particularly under conditions of perceived goal incongruence and insufficient resources. This type of appraisal captures users’ immediate understanding of the feasibility and value of the current usage situation, rather than a stable attitude toward the overall performance of the platform or system (Lazarus, 1991).

Although negative use evaluation is related to perceived usefulness, satisfaction, expectation confirmation, and information relevance, it is not equivalent to these constructs. Perceived usefulness usually refers to the general instrumental value of a system or information source, whereas satisfaction and expectation confirmation involve broader post-use evaluations. By contrast, negative use evaluation in this study captures a momentary judgment formed during a specific browsing episode: whether the current content still deserves continued cognitive and attentional investment.

Within the information-use context examined in this study, users are more likely to form threat-oriented rather than challenge-oriented evaluations. Under conditions of uncertain veracity and high redundancy, continued processing often appears effortful without providing clear informational benefits. As a result, sustained cognitive load is more likely to be interpreted as a stressful and low-value experience than as a manageable challenge that may yield meaningful returns (Lazarus, 1991; Lazarus and Folkman, 1984).

In information systems research, evaluative constructs are commonly operationalized in terms of perceived usefulness, satisfaction, or expectation confirmation and are widely used to explain users’ continued use or discontinuance behaviors. In the context of episodic discontinuation, however, treating use evaluation merely as an outcome-oriented attitudinal variable provides limited explanatory power for how cognitive strain is translated into emotional responses. From a cognitive appraisal perspective, evaluation is not a simple outcome of cognitive experience, but a process of meaning construction through which individuals interpret usage situations on the basis of cognitive load. As such, it plays a critical role in the transformation of cognitive experience into emotional response.

Building on this theoretical logic, prior research generally positions use evaluation after cognitive processing and before emotional response, conceptualizing it as a key psychological mechanism linking cognitive experience and affective outcomes. Along this appraisal-based pathway, negative use evaluation functions as a critical mediating stage through which experienced cognitive load is translated into emotional fatigue.

### 2.4 Emotional responses, emotional fatigue, and discontinuance intention

When users develop sustained negative evaluations of their usage situation, they become more vulnerable to negative emotional responses. Cognitive appraisal theory suggests that emotions emerge not from stimuli themselves but from how situations are interpreted in relation to one’s goals and coping capacity (Lazarus, 1991). In social media contexts, emotional reactions are therefore better understood as responses to evaluative judgments rather than direct reactions to information cues.

Empirical evidence indicates that persistent perceptions of high judgment costs and low returns increase feelings of anxiety, frustration, and psychological depletion (Bright et al., 2015; Liu et al., 2021). Emotional fatigue, in particular, captures the cumulative nature of such affective responses during sustained use.

In discontinuance research, emotional fatigue is often treated as a proximal antecedent of avoidance or temporary disengagement. However, it does not arise automatically from cognitive load alone. From a process perspective, fatigue develops when sustained cognitive burden is interpreted as devalued use. As such evaluations accumulate, users may regulate their affective state by reducing or temporarily suspending use.

Importantly, fatigue does not necessarily lead to permanent exit. Prior research suggests that users may oscillate between continued use and temporary suspension (Li et al., 2025). In episodic discontinuation contexts, emotional fatigue is therefore best understood as a state outcome whose behavioral consequences remain contingent on situational and regulatory factors.

### 2.5 Research gaps and focus of the present study

A synthesis of the existing literature indicates that prior research has largely succeeded in identifying a range of external pressure factors and their associations with discontinuance behavior. However, the continuous evolution of users’ internal psychological responses remains insufficiently theorized. In particular, within social media environments characterized by substantial variation in information quality, emotional fatigue is often treated as a given outcome, while its specific formation process remains a relative black box.

Against this background, we shift the analytical focus away from repeatedly verifying whether fatigue leads to discontinuance behavior. Instead, we move upstream to examine how differences in information quality gradually shape users’ emotional fatigue through a continuous psychological process involving cognitive load and use evaluation. At the same time, the study examines the relationship between emotional fatigue and self-reported episodic discontinuation intention at the within-subject level, in order to further clarify the boundary conditions under which this emotional state is associated with discontinuation-related intention.

## 3 Hypotheses development

### 3.1 Information quality cues and cognitive load

Cognitive load theory posits that individuals’ information processing is constrained by limited cognitive resources. When external information stimuli exceed individuals’ processing capacity in terms of quantity, complexity, or uncertainty, greater cognitive resources are required to complete comprehension and judgment tasks, resulting in higher levels of cognitive load (Sweller, 1988). In information-intensive social media environments, the cognitive processing burden faced by users arises not only from the sheer volume of information, but is also closely related to the quality characteristics of information content.

Prior research suggests that when information veracity is difficult to assess, structural cues are unclear, or content presentation is highly redundant, users are required to invest additional cognitive resources in filtering, integrating, and evaluating information, thereby substantially increasing information-processing costs (Eppler, 2004; Lang, 2000). Empirical studies have further shown that low-quality or highly uncertain information cues undermine processing efficiency, making individuals more likely to experience cognitive resource depletion or insufficiency (Liu et al., 2021).

Building on this body of research, the present study treats information veracity and information redundancy as key information quality cues that capture differences in cognitive processing demands under varying information conditions. Specifically, low-veracity information increases the cognitive costs associated with assessing information reliability, whereas high-redundancy information elevates the cognitive effort required for information filtering and integration. Both types of information quality cues are therefore expected to increase information-processing complexity and, in turn, elicit higher levels of cognitive load.

*H1:* Information quality cues (information veracity and information redundancy) will have significant effects on users’ cognitive load, such that low-veracity and high-redundancy information will lead to higher levels of cognitive load.

### 3.2 Cognitive load and negative use evaluation

Building on the appraisal framework discussed above, cognitive load does not automatically determine users’ responses to a usage situation. Rather, it shapes how users interpret the meaning of their cognitive experience. According to Cognitive Appraisal Theory (Lazarus, 1991; Lazarus and Folkman, 1984), primary appraisal involves evaluating situations in relation to personal goals, particularly in terms of goal relevance and goal congruence. Situations that remain goal-relevant but fail to support goal attainment are typically experienced as threatening.

In information-use contexts, sustained cognitive load may gradually undermine both perceived goal relevance and goal congruence. When effortful processing no longer advances users’ goals, the situation is more likely to be interpreted as burdensome rather than manageable. In this sense, negative use evaluation reflects a threat-oriented appraisal: continued engagement demands resources without adequately supporting intended objectives.

Importantly, this evaluation does not represent a stable attitude toward the platform. Instead, it captures a situational judgment about whether the current usage context remains worthwhile. Prior research similarly shows that when users perceive high investment costs alongside limited returns, unfavorable usage evaluations are more likely to emerge (Bhattacherjee, 2001; Venkatesh et al., 2012).

From this perspective, higher levels of cognitive load are expected to increase negative use evaluation.

*H2:* Higher levels of cognitive load will be associated with stronger negative use evaluation.

### 3.3 Negative use evaluation and emotional fatigue

Cognitive appraisal theory further suggests that emotional responses are not direct reflections of external stimuli or cognitive experiences, but instead arise from individuals’ subjective evaluations of situational meaning (Lazarus, 1991). When users develop sustained negative evaluations during social media use, such as perceiving continued use as no longer worthwhile or as exceeding their coping capacity, these evaluative judgments provide the psychological basis for the emergence of negative emotional experiences. Prior research has likewise shown that negative attitudes and unfavorable use evaluations are significantly associated with emotional exhaustion and social media fatigue (Bright et al., 2015; Zhang et al., 2020).

Within the context of the study, emotional fatigue is conceptualized as a subjective affective state that develops from the accumulation of sustained cognitive load and negative usage experiences, rather than as an immediate behavioral response. Accordingly, when users form higher levels of negative use evaluation toward a usage situation, they are more likely to experience stronger emotional fatigue.

*H3:* Stronger negative use evaluation will be associated with higher levels of emotional fatigue.

### 3.4 Emotional fatigue and episodic discontinuation intention

Prior research has commonly treated emotional fatigue as an important psychological antecedent of user discontinuance or usage interruption (Dhir et al., 2018; Maier et al., 2015). However, unlike permanent exit, episodic discontinuation is more likely to function as a short-term emotion-regulation or self-protective strategy, whose occurrence is jointly shaped by specific usage contexts and individual differences (Li et al., 2025). Accordingly, whether—and to what extent—emotional fatigue translates into episodic discontinuation behavior that remains an open empirical question requiring more nuanced investigation.

Importantly, our study does not position this relationship as a core hypothesis. This is because, under short-term task conditions, participants may not readily form strong discontinuation intentions, and the corresponding effects are expected to be less stable. Therefore, we treat the relationship between emotional fatigue and episodic discontinuation as a supplementary test rather than as a central component of the proposed psychological process model.

*RQ:* At the within-subject level, does emotional fatigue reliably translate into episodic discontinuation intention?

## 4 Research methods

To systematically examine how information quality–related features influence users’ information-processing processes as well as their subsequent psychological responses and self-reported discontinuation intention, this study combines experimental manipulation with questionnaire-based measurement and adopts a within-subject experimental design to obtain multilevel data. Given the clearly hierarchical structure of the experimental data—namely, repeated observations nested within individuals—the subsequent statistical analyses primarily employ linear mixed-effects models (LMMs) to account for between-subject variability and to enhance the robustness of the estimates.

### 4.1 Research design

#### 4.1.1 Experimental design

This study employed a 2 × 2 within-subject experimental design, with information veracity (high vs. low, with low-veracity texts implemented by introducing inaccurate or unverified information) and information redundancy (low vs. high) as the two core manipulated factors. This design yielded four experimental conditions: high veracity × low redundancy, high veracity × high redundancy, low veracity × low redundancy, and low veracity × high redundancy. This design simulates how users’ cognitive and psychological responses vary under different levels of information reliability and uncertainty commonly found in online environments.

Social interaction complexity and platform functional complexity were treated solely as background contextual factors and were not included in the main analytical pathway. By manipulating information veracity and redundancy, we focus on examining how information quality–related features influence cognitive processing, use evaluation, emotional experience, and episodic discontinuation intention.

#### 4.1.2 Experimental materials

The experimental stimuli consisted of web-based travel guide materials designed in the style of Xiaohongshu. Each participant completed four experimental tasks sequentially, corresponding to the four information conditions. The experimental procedure followed a basic sequence of stimulus exposure–immediate measurement–task switching. During the stimulus presentation stage, participants read the web-based materials displayed on a computer screen. Immediately after each task, participants completed the corresponding questionnaire measures assessing cognitive load, negative use evaluation, emotional fatigue, and episodic discontinuation intention.

### 4.2 Survey design and measures

The experimental materials consisted of travel guide posts for Yunnan Province, developed from authentic content commonly found on the Xiaohongshu platform. Four texts with comparable structures were constructed around the same itinerary, differing only in information veracity and information redundancy in a 2 × 2 within-subject design. In this study, these two dimensions were chosen to capture complementary aspects of information quality in social media browsing. Information veracity refers to the perceived accuracy and reliability of content. Information redundancy, by contrast, reflects the extent of repetitive or excessive information that increases cognitive processing demands. Compared with other dimensions such as completeness or relevance, these two can be manipulated through localized adjustments without altering the core content, which helps maintain experimental control and comparability across conditions.

Although redundancy was partly operationalized through increased textual volume, the manipulation was intended to capture repetitive informational content rather than topic expansion. High-redundancy materials repeated or paraphrased information already presented, without adding new decision-relevant facts. Across conditions, the topic, itinerary structure, visual layout, number of images, task instructions, and emotional tone were kept consistent. The low- and high-redundancy versions mainly differed in the degree of repetitive elaboration. A summary of the stimulus design is provided in Supplementary Appendix B, and the complete webpage-based experimental stimuli are provided as an online Supplementary file.

High-veracity texts were based on verified reports and official tourism information, whereas low-veracity texts introduced inaccurate or unverified fragments while keeping tone, structure, and style consistent. Low-redundancy texts focused on core information (approximately 280 words). High-redundancy texts expanded to about 580 words by adding repetitive descriptions, without changing the underlying facts. Topic scope, tone, layout, and image presentation were kept consistent across all conditions.

To minimize potential confounding effects, identical reading windows and task pacing were applied. A pretest with 20 participants confirmed the effectiveness of the manipulations. Perceived veracity and redundancy were measured using three-item scales (5-point Likert), showing acceptable reliability (*α* = 0.86 and 0.81). Independent-samples t-tests confirmed significant differences between conditions for both perceived veracity, t(18) = 8.47, *p* < 0.001, and perceived redundancy, t(18) = 8.16, *p* < 0.001.

To reduce participant burden, manipulation check items were not repeated in the formal experiment. Instead, veracity and redundancy were treated as experimental condition variables in the subsequent analyses (Table 1).

**Table 1:** A separate pilot sample (*N* = 20) was used to conduct the manipulation check.

Variable	Condition	Cronbach’s *α*	M	SD
Information veracity	High	0.86	4.30	0.50
	Low		2.10	0.65
Information redundancy	High	0.81	4.10	0.55
	Low		2.05	0.60

Source: Authors’ own work.

### 4.3 Participants and procedure

A total of 123 participants were recruited for the experiment through campus announcements and experimental volunteer channels. The sample included 55 males and 68 females, with ages ranging from 20 to 29 years (*M* = 22.3, SD = 1.8). All participants were undergraduate or graduate students who were familiar with common mobile internet platforms such as Xiaohongshu, Douyin, and Weibo, and had basic experience in online information browsing and evaluation. All participants had normal or corrected-to-normal vision and reported no history of serious visual impairments or eye-related diseases.

Participation was voluntary, and all participants received appropriate compensation. Prior to the experiment, participants provided written informed consent. The research protocol was approved by the institutional ethics committee, and all experimental data were collected anonymously and used solely for academic research purposes. The experiment was conducted in the university’s behavioral laboratory under one-on-one guidance by the experimenter.

Before the formal task began, the experimenter briefly introduced the experimental procedure and key instructions. Participants then completed a short practice task to familiarize themselves with the experimental interface and task operations. During the formal experiment, each participant completed four browsing tasks corresponding to the four experimental conditions of information veracity and information redundancy. The order of conditions was counterbalanced using a Latin square design to reduce order and fatigue effects.

Each condition followed a stimulus exposure–immediate measurement procedure. Stimulus exposure refers to the stage in which participants viewed one Xiaohongshu-style travel-guide post displayed on a computer screen. Immediately after viewing each post, participants completed questionnaire measures assessing cognitive load, negative use evaluation, emotional fatigue, and episodic discontinuation intention for that condition. Short breaks were provided between conditions. The entire experimental session lasted approximately 20 min, including instructions and practice.

Undergraduate and graduate students were selected for several reasons. First, university students are frequent users of social media and have relatively rich experience in online information browsing and evaluation, which enabled them to complete the experimental tasks effectively. Second, as an initial test of the proposed psychological process model, a relatively homogeneous sample helped reduce potential confounding effects and improve internal validity. Finally, this sample provides a useful baseline for future replication studies involving more diverse populations, such as older adults or individuals with different educational backgrounds.

### 4.4 Measurement of variables

Information quality differences were implemented through experimental manipulation and treated as measured condition variables (high vs. low information veracity; low vs. high information redundancy). The effectiveness of the material manipulations was verified through a pretest conducted prior to the formal experiment. The primary variables measured in the formal experiment included cognitive load, negative use evaluation, emotional fatigue, and episodic discontinuation intention.

Cognitive load was used to reflect participants’ perceived difficulty in understanding information, perceived information complexity, and the consumption of attentional resources during information browsing. The scale was adapted from the multidimensional cognitive load questionnaires proposed by Klepsch et al. (2017) and Leppink et al. (2013). In accordance with the characteristics of the experimental tasks in the present study, items closely related to external information structure complexity and information comprehension costs were retained, resulting in a total of 4 measurement items. This scale has been widely applied in educational psychology and media cognition research and has demonstrated good reliability in prior studies (with Cronbach’s *α* typically exceeding 0.80).

Negative use evaluation was employed to capture users’ immediate cognitive judgments regarding whether the current information content was worth continued attentional investment during browsing. This construct is grounded in the primary appraisal stage of Cognitive Appraisal Theory and reflects users’ evaluative interpretation of whether the current information environment remains worthwhile and goal-congruent during ongoing browsing. To more precisely capture the evaluative meaning of goal incongruence and reduced situational value, four items were developed around two key dimensions of primary appraisal—goal congruence and goal relevance—reflecting whether the information supports users’ current goals and meets their practical information needs. All items were measured using five-point Likert scales. Importantly, the study focuses on situational, momentary cognitive evaluations formed during use, rather than on global attitudes toward the platform or system.

Although conceptually related to constructs such as perceived usefulness, expectation confirmation, and information relevance, the present construct differs in several important respects. Rather than evaluating the overall usefulness or quality of a platform or information system, negative use evaluation in this study captures users’ momentary interpretation of whether continued engagement remains worthwhile within an ongoing browsing episode.

Emotional fatigue, as the key affective outcome variable, refers to participants’ situational fatigue experience arising during the current information-browsing task. Drawing on established scales from prior social media fatigue research, emotional fatigue was measured using 4 Likert-type items assessing participants’ overall affective experience during information browsing. Immediate emotional reactions such as anxiety were measured only as supplementary indicators to illustrate potential negative emotional responses induced by high cognitive load and were not included in the core analytical model.

Following established definitions of non-continuous use and episodic discontinuation in social media research (Dhir et al., 2018; Li et al., 2025; Maier et al., 2015), episodic discontinuation was operationalized as participants’ intention to temporarily suspend or avoid continued use within the current information-browsing context. This operationalization emphasizes self-reported, context-triggered episodic discontinuation intention, reflecting a non-permanent tendency toward usage interruption rather than long-term or complete exit behavior.

Episodic discontinuation intention captures individuals’ stage-specific tendency to interrupt usage while browsing social media content as a result of subjective fatigue or psychological burden. The present study focuses on immediately triggered discontinuance motivation within the usage context, rather than permanent platform abandonment. It should be noted that the measured intention does not represent actual discontinuance behavior, but rather reflects participants’ subjective readiness to temporarily discontinue browsing in the current situation. Prior research indicates that usage fatigue often motivates regulation of continued use or stage-based interruption (Luqman et al., 2017; Luqman et al., 2020). Three items were measured using five-point Likert scales, with higher scores indicating stronger episodic discontinuation intention (Table 2).

**Table 2:** Measurement items of variables.

Variable	No	Item	Reference
Cognitive load	CL1	This information makes me feel very confused.	Leppink et al. (2013); Klepsch et al. (2017); Paas (1992)
	CL2	I need to spend a lot of effort to understand this information.	
	CL3	I often forget the content I have read before during browsing.	
	CL4	I need to spend a lot of effort to screen which information is more important.	
Negative use evaluation	NUE1	I feel that these posts are not very helpful for me to plan my trip to Yunnan.	Lazarus and Folkman (1984); Cuervo (2006)
	NUE2	I did not get the information I really needed when browsing this content.	
	NUE3	The information in these posts is not very consistent with the travel questions I care about.	
	NUE4	After reading this content, I am not clearer about my travel plan.	
Emotional fatigue	EF1	Using social media makes me feel emotionally exhausted.	Maier et al. (2015); Bright et al. (2015)
	EF2	I feel that my energy is consumed when using social media.	
	EF3	I feel tired of continuing to use social media.	
	EF4	I feel emotionally drained when browsing these posts.	
Episodic discontinuation intention	EDI2	I have the idea of not wanting to continue reading in depth during browsing.	Luqman et al. (2017); Luqman et al. (2020)
	EDI3	I hope to temporarily stop browsing when browsing.	
	EDI4	When the content of the post brings a sense of burden, I tend to interrupt browsing.	

Source: Authors’ own work.

### 4.5 Data analysis method

An *a priori* power analysis conducted using G*Power 3.1 indicated that, with an alpha level of 0.05 and an expected medium effect size (*f* = 0.25), a sample size of approximately *N* = 128 would be required to achieve a statistical power of 0.80. The actual sample size of the study (*N* = 123) was close to this threshold, suggesting that the statistical power was generally adequate.

Given the within-subject experimental design and the nested structure of the data (i.e., repeated observations nested within individuals), mixed-effects modeling was employed for data analysis. For continuous dependent variables—including cognitive load, negative use evaluation, emotional fatigue, and episodic discontinuation intention—linear mixed-effects models (LMMs) were used.

Information veracity, information redundancy, and their interaction were specified as fixed effects, while participant ID was included as a random intercept to account for between-subject variability. Alternative random-effects structures were explored, but models including random slopes showed convergence instability; therefore, random-intercept models were retained to ensure stability and interpretability.

Descriptive statistics, reliability analyses, factor analyses, and the main linear mixed-effects models were conducted in SPSS 20. The supplementary sequential indirect-effect analysis, which examined the full chain from cognitive load through negative use evaluation and emotional fatigue to episodic discontinuation intention, was conducted in Python using OLS-based path models with participant-level cluster bootstrap. Detailed model specifications and analytical results are reported in Section 5.

## 5 Results

### 5.1 Descriptive statistics, reliability, and measurement validity

All subjective scales were adapted from established instruments and adjusted to fit the experimental context. The four constructs showed acceptable internal consistency, with Cronbach’s *α* values of 0.935, 0.827, 0.886, and 0.777 for cognitive load, negative use evaluation, emotional fatigue, and episodic discontinuation intention, respectively. The exploratory factor loading matrix is reported in Supplementary Appendix C. Additional checks also supported measurement validity: standardized item loadings were close to or above 0.70, CR values ranged from 0.865 to 0.946, and AVE values across the four constructs ranged from 0.618 to 0.813. Discriminant validity was supported by the Fornell-Larcker criterion.

Based on this, descriptive statistics were first conducted to summarize the overall distributional characteristics of the main variables and to provide an overview of users’ psychological responses under different information conditions. The means and standard deviations of all variables across experimental conditions are reported in Table 3.

**Table 3:** Means and standard deviations of variables.

Variable	High veracity × low redundancy		High veracity × high redundancy		Low veracity × low redundancy		Low veracity × high redundancy	
	M	SD	M	SD	M	SD	M	SD
Cognitive load	2.35	0.44	2.89	0.52	2.98	0.33	3.39	0.48
Negative use evaluation	2.38	0.80	2.91	0.88	3.26	1.06	3.98	0.88
Emotional fatigue	2.88	0.75	3.67	0.65	4.10	0.75	3.32	0.98
Episodic discontinuation Intention	2.58	0.81	3.04	0.76	3.54	1.00	3.98	0.75

High veracity × low redundancy serves as the reference condition.

Cognitive load values represent averaged scale scores within each condition, which reduces dispersion.

Source: Authors’ own work.

Based on the descriptive results, cognitive load, negative use evaluation, emotional fatigue, and episodic discontinuation intention exhibited broadly consistent patterns across different information quality conditions. Compared with the high-veracity and low-redundancy condition, the mean levels of these variables were generally higher under conditions characterized by low veracity and high redundancy. This pattern suggests that differences in information quality had already exerted an observable influence on users’ information-processing experiences and psychological responses at the descriptive level.

### 5.2 Effects of information quality cues on subjective experience

To further examine whether the experimentally manipulated information quality cues exert direct effects on users’ subjective experiences, linear mixed-effects models (LMM) were employed. Specifically, the effects of information veracity and information redundancy on users’ cognitive load, negative use evaluation, and emotional fatigue were examined (Tables 4–6).

**Table 4:** Effects of information veracity and redundancy on cognitive load (LMM).

Effect	*β*	SE	*t*	*p*	95% CI
Intercept (high veracity × low redundancy)	2.352	0.041	57.90	< 0.001	[2.272, 2.431]
Veracity (low vs. high)	0.630	0.057	10.97	< 0.001	[0.517, 0.743]
Redundancy (high vs. low)	0.543	0.057	9.45	< 0.001	[0.430, 0.656]
Veracity × redundancy	−0.130	0.081	−1.60	0.110	[−0.290, 0.030]

Intercept represents the estimated mean under the reference condition.

Source: Authors’ own work.

**Table 5:** Effects of information veracity and redundancy on negative use evaluation (LMM).

Effect	*β*	SE	*t*	*p*	95% CI
Intercept (high veracity × low redundancy)	2.382	0.082	29.13	*p* < 0.001	[2.222, 2.542]
Veracity (low vs. High)	0.876	0.115	7.65	*p* < 0.001	[0.652, 1.100]
Redundancy (high vs. Low)	0.524	0.115	4.58	*p* < 0.001	[0.300, 0.749]
Veracity × redundancy	0.199	0.162	1.23	*p* = 0.219	[−0.118, 0.517]

Intercept represents the estimated mean under the reference condition.

Source: Authors’ own work.

**Table 6:** Effects of information veracity and redundancy on emotional fatigue (LMM).

Effect	*β*	SE	*t*	*p*	95% CI
Intercept (high veracity × low redundancy)	2.884	0.071	40.38	*p* < 0.001	[2.744, 3.024]
Veracity (low vs. high)	1.220	0.101	12.07	*p* < 0.001	[1.022, 1.417]
Redundancy (high vs. low)	0.791	0.101	7.83	*p* < 0.001	[0.593, 0.989]
Veracity × redundancy	−1.575	0.143	−11.03	*p* < 0.001	[−1.855, −1.295]

Intercept represents the estimated mean under the reference condition.

Source: Authors’ own work.

In these models, information veracity and information redundancy were specified as fixed effects, while participant ID was included as a random effect to control for individual differences.

#### 5.2.1 Effects on cognitive load

**Figure 1:**
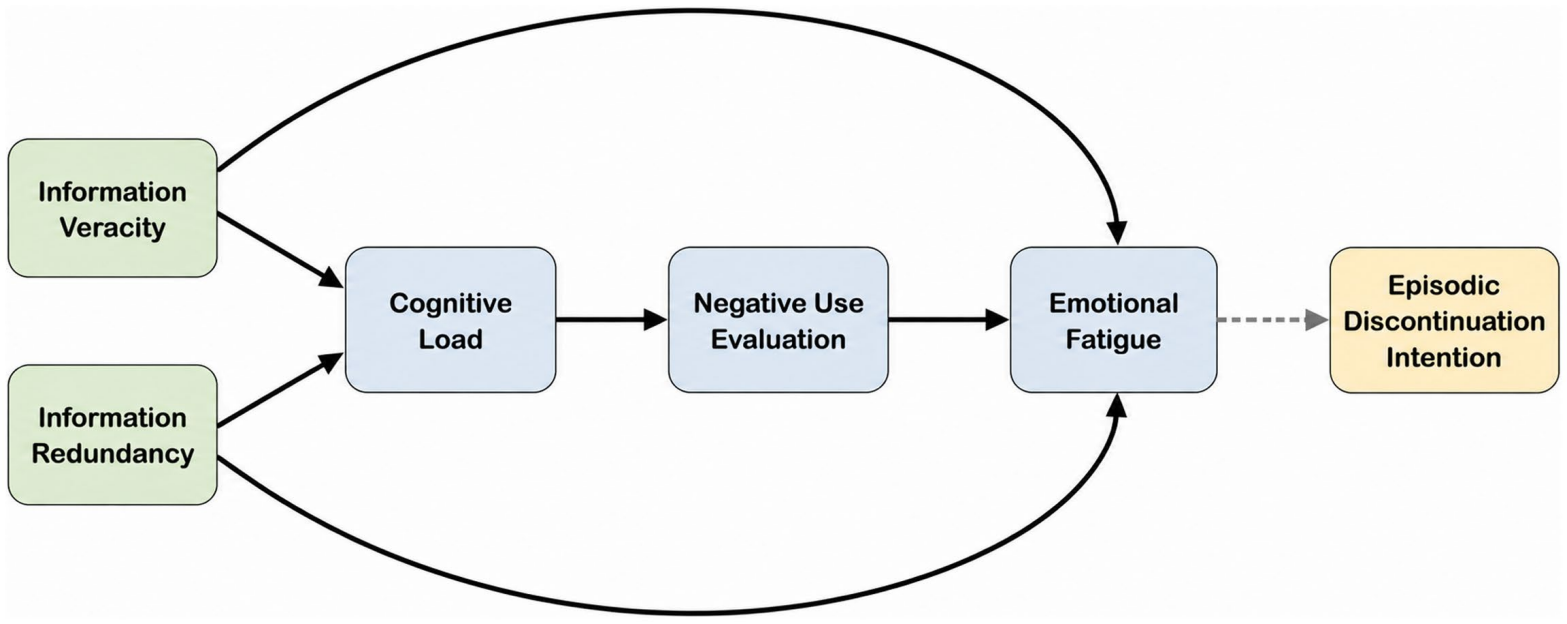
Experimental design and analytical framework. Source: Authors’ own work.

**Figure 2:**
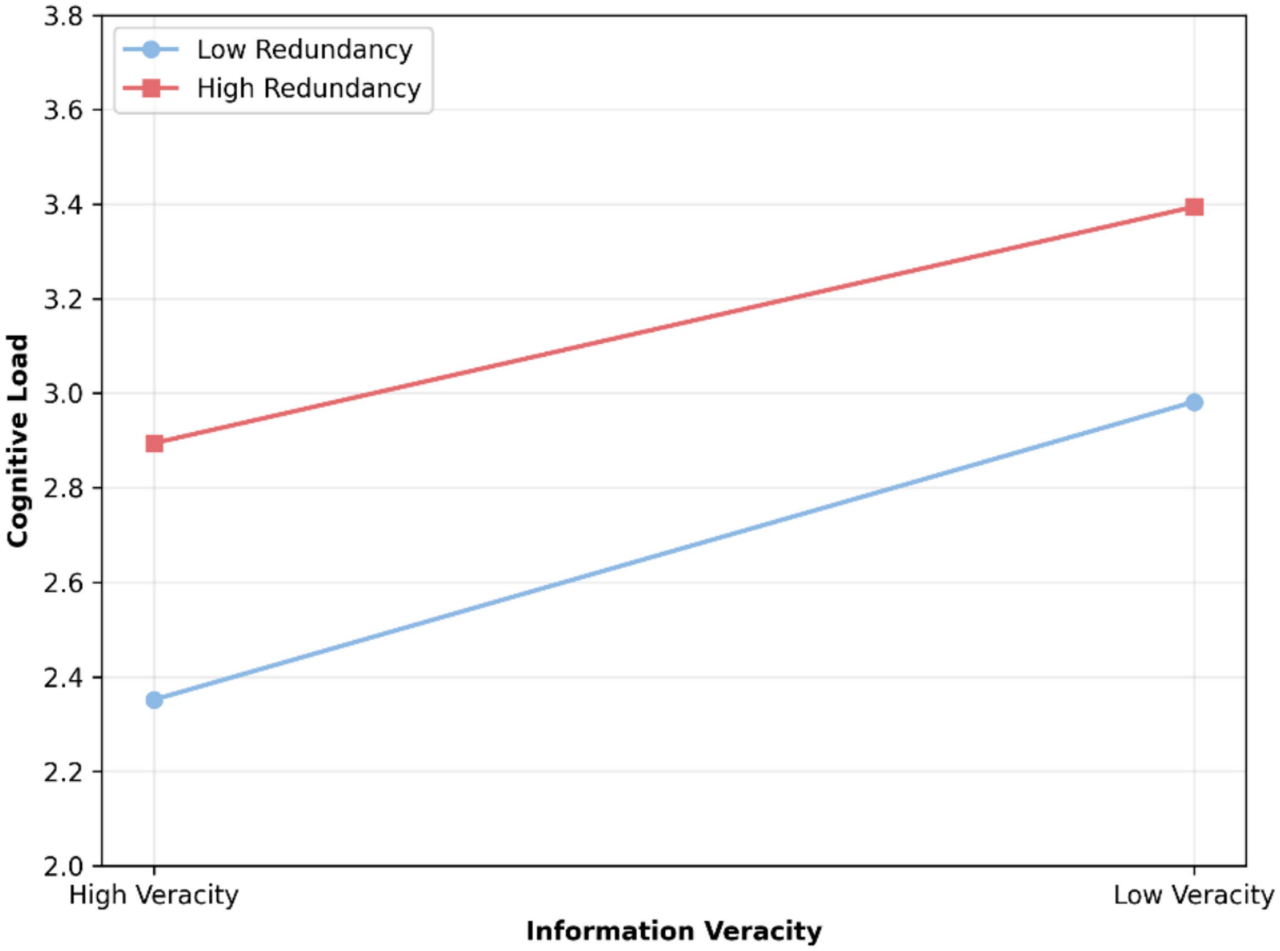
Estimated marginal means of cognitive load across experimental conditions. Source: Authors’ own work.

**Figure 3:**
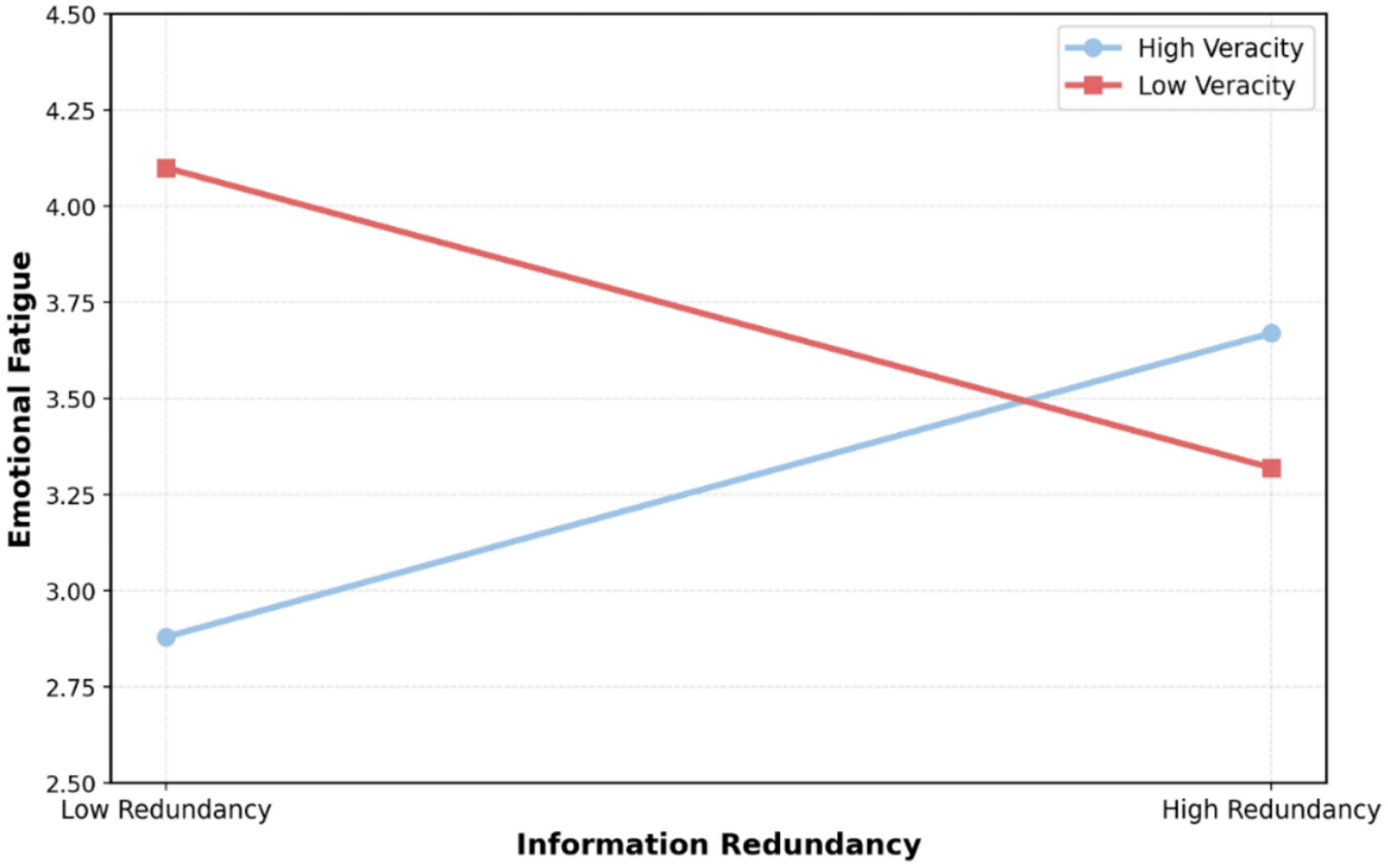
Interaction effects on emotional fatigue. Source: Authors’ own work.

To examine the effects of information veracity and information redundancy on individuals’ cognitive load, a linear mixed-effects model (LMM) was estimated with mean cognitive load as the dependent variable. Information veracity (high vs. low) and information redundancy (low vs. high) were specified as fixed effects, and participant ID was included as a random effect. The model included random intercepts only, in order to control for baseline differences across participants. For the categorical predictors, the high-veracity and low-redundancy conditions were specified as the reference categories (Figures 1–3).

The LMM results revealed a significant main effect of information veracity on cognitive load. Compared with high-veracity information, low-veracity information significantly increased participants’ cognitive load (*β* = 0.630, SE = 0.057, *p* < 0.001). Information redundancy also exhibited a significant main effect, such that high-redundancy information led to significantly higher cognitive load than low-redundancy information (*β* = 0.543, SE = 0.057, *p* < 0.001).

By contrast, the interaction effect between information veracity and information redundancy did not reach statistical significance (*β* = −0.130, SE = 0.081, *p* = 0.110). This result indicates that the two information-quality cues primarily exerted independent effects on cognitive load. In other words, low veracity and high redundancy each increased users’ information-processing burden, but their combined effect did not provide clear evidence of an additional interactive influence at the cognitive-load stage.

Based on the model estimates, the predicted means of cognitive load across experimental conditions exhibited a clear gradient pattern. Cognitive load was lowest under the high-veracity and low-redundancy condition (*M* = 2.35). When either high redundancy or low information veracity was present alone, cognitive load increased to *M* = 2.89 and *M* = 2.98, respectively. When low veracity and high redundancy co-occurred, cognitive load reached its highest level (*M* = 3.39). These results indicate that both information veracity and information redundancy increased individuals’ information-processing burden.

Overall, both information veracity and information redundancy significantly elevated the level of cognitive load experienced during information processing. However, their interaction did not reach statistical significance, suggesting that the two information-quality cues primarily exerted independent effects on cognitive load. Taken together, these findings provide support for H1.

#### 5.2.2 Effects on negative use evaluation

To examine the effects of information veracity and information redundancy on users’ negative use evaluation, a linear mixed-effects model (LMM) was estimated with negative use evaluation as the dependent variable. Information veracity (high vs. low) and information redundancy (low vs. high) were specified as fixed effects, and participant ID was included as a random intercept to control for between-subject differences. The high-veracity × low-redundancy condition was set as the reference category.

The results revealed a significant main effect of information veracity on negative use evaluation (*β* = 0.876, SE = 0.115, *t* = 7.65, *p* < 0.001). Compared with high-veracity information, low-veracity information significantly increased participants’ negative use evaluation, indicating that when information credibility decreases, users are more likely to form unfavorable judgments about the current content.

Information redundancy also exhibited a significant main effect (*β* = 0.524, SE = 0.115, *t* = 4.58, *p* < 0.001). Compared with low-redundancy information, high-redundancy information significantly strengthened negative use evaluation, suggesting that redundant information amplifies users’ negative perceptions and judgments of diminished value during use.

By contrast, the interaction effect between information veracity and information redundancy did not reach statistical significance (*β* = 0.199, SE = 0.162, *t* = 1.23, *p* = 0.219). This result indicates that, in the present experimental context, the effects of veracity and redundancy on negative use evaluation are primarily manifested as relatively independent direct effects rather than through their combined interaction.

It should be noted that negative use evaluation in this study does not refer to users’ overall satisfaction with the platform or system. Instead, it is operationalized as a form of situational devaluation, capturing users’ momentary judgments regarding whether the current content is worth continued attentional and cognitive investment within a specific information-use context, rather than a summary evaluation of the system’s overall functionality or value.

#### 5.2.3 Effects on emotional fatigue

A linear mixed-effects model (LMM) was estimated with emotional fatigue as the dependent variable. Information veracity (high vs. low) and information redundancy (low vs. high) were specified as fixed effects, and participant ID was included as a random effect. The high-veracity × low-redundancy condition was set as the reference category.

The results revealed a significant main effect of information veracity on emotional fatigue, such that low-veracity information was overall associated with higher levels of emotional fatigue (*β* = 1.220, SE = 0.101, *p* < 0.001). Information redundancy also exhibited a significant main effect, with high-redundancy information increasing users’ emotional fatigue at the overall level (*β* = 0.791, SE = 0.101, *p* < 0.001).

In addition, a significant interaction effect between information veracity and information redundancy on emotional fatigue was observed (*β* = −1.575, SE = 0.143, *p* < 0.001). Examination of the conditional means further showed that under low-redundancy conditions, emotional fatigue was substantially higher in the low-veracity condition than in the high-veracity condition (4.10 vs. 2.88). By contrast, under high-redundancy conditions, the difference in emotional fatigue between the two veracity conditions was markedly reduced (3.32 vs. 3.67), with fatigue experiences converging across veracity levels and exhibiting a pattern of attenuation.

### 5.3 From cognitive load to negative use evaluation

In the previous section, this study examined the direct effects of information veracity and information redundancy on negative use evaluation from the perspective of information content characteristics. The results showed that information quality cues alone were sufficient to significantly alter users’ value judgments of the usage situation. However, from the perspective of Cognitive Appraisal Theory, the mere presence of information cues is not sufficient to explain how evaluative judgments are formed. Evaluation is not a simple reaction to external stimuli, but rather an interpretive outcome grounded in individuals’ subjective processing experiences. Accordingly, we further examine whether cognitive load plays an independent role in shaping evaluative judgments from the perspective of information-processing experience.

After establishing that information veracity and information redundancy significantly alter users’ cognitive processing burden, we further tested whether cognitive load influences users’ evaluative judgments of the current information-use situation. A linear mixed-effects model (LMM) was estimated with the mean score of negative use evaluation as the dependent variable and mean cognitive load as the predictor. Participant ID was included as a random intercept to control for baseline differences across individuals.

The model estimates indicated that, after accounting for within-subject repeated measures and individual differences, cognitive load exerted a significant positive effect on negative use evaluation (*β* = 1.237, SE = 0.062, *t* = 19.90, *p* < 0.001, 95% CI [1.114, 1.359]). This result suggests that as the level of cognitive load experienced during information processing increases, individuals are more likely to form unfavorable evaluations of the current information-use situation, perceiving the content as failing to effectively support their goals or as no longer worth sustained attentional and cognitive investment.

From the perspective of Cognitive Appraisal Theory, cognitive load itself does not directly constitute a value judgment about the information situation; rather, it represents a subjective experience that has not yet been assigned explicit meaning. Only when individuals interpret this processing experience through evaluative appraisal does cognitive load translate into a directional judgment. Accordingly, in the present study, the role of cognitive load is primarily reflected in its significant predictive effect on use evaluation, rather than in a direct influence on subsequent emotional or behavioral responses. These results support H2.

### 5.4 From negative use evaluation to emotional fatigue

After establishing that cognitive load exerts a significant influence on use evaluation, the analysis turns to whether evaluative judgments are associated with affective responses. A linear mixed-effects model (LMM) was estimated with emotional fatigue as the dependent variable and the mean score of negative use evaluation as the predictor.

The results showed that negative use evaluation had a significant positive effect on emotional fatigue [*β* = 0.248, SE = 0.036, *t* = 6.81, *p* < 0.001, 95% CI (0.177, 0.319)]. When the information-browsing experience is persistently appraised as no longer worth continued investment, or when there is little perceived benefit to continued engagement, emotional exhaustion and fatigue become more likely.

This finding is consistent with a core proposition of Cognitive Appraisal Theory: emotional responses do not directly reflect cognitive load itself, but instead arise from subjective interpretations of situational meaning. Within the context of the present study, negative use evaluation operates as a key evaluative process through which cognitive processing experiences are translated into emotional fatigue. These findings provide support for H3.

### 5.5 Emotional fatigue and episodic discontinuation intention

To examine the effect of emotional fatigue on episodic discontinuation intention, a linear mixed-effects model (LMM) was estimated with participant ID specified as a random intercept. The results indicated that, after controlling for individual differences, emotional fatigue exerted a significant positive effect on episodic discontinuation intention [*β* = 0.385, SE = 0.046, *t* = 8.41, *p* < 0.001, 95% CI (0.295, 0.475)].

These findings indicate that even in an experimental context where actual discontinuance behavior was not observed and the outcome was measured as self-reported intention, affective fatigue experiences are sufficient to significantly influence individuals’ subjective evaluations of whether to continue information use, thereby manifesting as stronger episodic discontinuation intention. In response to the research question, emotional fatigue reliably translates into episodic discontinuation intention at the within-subject level.

### 5.6 Sequential process analysis

In the preceding analyses, the relationships among cognitive load, negative use evaluation, emotional fatigue, and episodic discontinuation intention were examined step by step. To further clarify how the proposed psychological process unfolds across different stages of browsing, we conducted an additional analysis to examine the full sequential pathway linking information quality cues to self-reported episodic discontinuation intention. Given the repeated-measures structure of the data, the sequential indirect effects were estimated using OLS-based path models with participant-level cluster bootstrap in Python. Specifically, 5,000 bootstrap resamples were performed. In each resample, participants were sampled with replacement, and all repeated observations belonging to each sampled participant were retained, thereby preserving the within-subject data structure.

The results suggest that the effects of information veracity and information redundancy on episodic discontinuation intention do not emerge in an isolated or immediate manner. Instead, their influence unfolds progressively through cognitive load, negative use evaluation, and emotional fatigue. More specifically, low-veracity information increases users’ information-processing burden, which subsequently strengthens negative use evaluation toward the current browsing experience. When users begin to perceive continued attentional investment as no longer worthwhile, emotional fatigue further accumulates and eventually translates into stronger episodic discontinuation intention. The sequential indirect effect of information veracity through cognitive load, negative use evaluation, and emotional fatigue was significant [Effec*t* = 0.042, 95% Bootstrap CI (0.02261, 0.06737)]. A similar pattern was observed for information redundancy, whose sequential indirect effect through the same pathway was also significant [Effec*t* = 0.036, 95% Bootstrap CI (0.01924, 0.05623)].

These supplementary findings further indicate that episodic discontinuation is not an immediate response to problematic information cues alone. Rather, it gradually develops during browsing through a continuous psychological process. Information quality cues may initially increase processing burden, but cognitive burden itself is not sufficient to directly produce self-reported episodic discontinuation intention. Only when this burden is interpreted as a low-value or unrewarding usage experience does it gradually evolve into emotional fatigue and subsequently increase discontinuation intention. In this sense, the mechanism identified in the present study is better understood as a staged process involving cognitive burden, evaluative interpretation, and emotional accumulation, rather than as a simple stimulus–response relationship.

### 5.7 Summary of results

Taken together, the above analyses indicate that differences in information quality can significantly shape users’ emotional fatigue through a series of sequential psychological processes. Specifically, low information veracity and high information redundancy first increase users’ information-processing burden, which in turn fosters negative evaluations of the usage situation. However, at the emotional stage, their effects were not fully additive, as high redundancy partly attenuated the differentiating role of veracity in shaping emotional fatigue. As these negative evaluations accumulate over time, they gradually develop into emotional fatigue, which further increases episodic discontinuation intention.

## 6 Discussion

Adopting a psychological process perspective, this study examines how differences in information quality gradually shape emotional fatigue through cognitive load and use evaluation, and further tests—at the within-subject level—whether emotional fatigue is associated with episodic discontinuation intention. In contrast to prior research that has primarily explained discontinuance by focusing on external stressors or emotional outcomes, this research does not focus on re-establishing the well-documented link between fatigue and discontinuance. Instead, the analytical focus is shifted earlier in the usage episode to the formation process of emotional fatigue itself, empirically clarifying how information-quality cues are translated into short-term discontinuation-related intention.

The core contribution of this work lies in demonstrating that the impact of information quality on emotional fatigue is not direct. Rather, fatigue emerges only after processing load is cognitively interpreted as not worth the effort through a threat-oriented evaluative interpretation. By specifying this appraisal stage, the study sheds light on what occurs within the “Organism” component of the SOR framework.

### 6.1 From cognitive load to evaluation

In information systems and continuance research, evaluation-related constructs such as perceived usefulness, satisfaction, and expectation confirmation are often treated as important predictors of continued use or discontinuance (Bhattacherjee, 2001; Venkatesh et al., 2012). By contrast, in studies of social media fatigue and discontinuance, evaluative variables are more often treated in a simplified way, or absorbed into broader outcome-oriented attitudes, with relatively little attention given to how they emerge from cognitive processing itself (Bright et al., 2015; Zhang et al., 2020).

The findings of the present study suggest that higher cognitive load is associated with stronger negative use evaluation. In other words, users do not move directly from processing burden to emotional response. Before fatigue takes shape, there is an intervening stage in which individuals assess whether continued investment of attention and effort still feels worthwhile. Importantly, the construct examined here should not be interpreted as a direct measure of threat appraisal itself, but rather as a situational evaluative interpretation formed under perceived goal incongruence during ongoing information use. This reading is consistent with the central logic of Cognitive Appraisal Theory, which emphasizes that emotional responses are shaped not simply by external conditions or internal strain, but by how those experiences are interpreted in relation to one’s goals and situational meaning (Lazarus, 1991; Lazarus and Folkman, 1984).

This point matters for discontinuance research. If cognitive load and emotion are placed side by side without considering this evaluative step, it becomes harder to explain how burdens created by information quality are subjectively interpreted and then carried forward into later psychological outcomes. What the present findings suggest is that negative use evaluation is not just an additional variable in the chain. It is the stage at which cognitive burden begins to acquire directional meaning.

### 6.2 The formation of emotional fatigue

Prior research has often treated emotional fatigue, or social media fatigue, as the product of prolonged exposure to complex information environments and persistent social demands. It has also consistently linked fatigue to avoidance behaviors and discontinuance tendencies (Dhir et al., 2018; Maier et al., 2015). The results of the present study point to a more gradual process. Negative use evaluation shows a significant positive effect on emotional fatigue, suggesting that fatigue does not arise immediately from a single episode of cognitive strain or from isolated information cues alone. Instead, it develops over time as users repeatedly interpret their browsing experience as unrewarding, inefficient, or no longer worth sustained effort. This interpretation is consistent with Lazarus (1991) view that emotional responses are grounded in meaning appraisal rather than produced automatically by external stimuli.

Seen in this way, emotional fatigue is not simply the direct result of being exposed to problematic information. It is also shaped by how that experience is understood. This helps explain why users facing seemingly similar information environments may still report very different levels of fatigue (Bright et al., 2015). What matters is not only the information itself, but also how its value, usefulness, and manageability are continually reassessed as browsing unfolds.

### 6.3 From emotional fatigue to episodic discontinuation intention

A substantial body of research has identified emotional fatigue as an important proximal antecedent of non-continuous use and discontinuance (Dhir et al., 2018; Maier et al., 2015). Much of this evidence, however, has come from between-subject comparisons or from more aggregated indicators of use. In short-duration experimental settings, where exit costs are relatively low and actual withdrawal is not easily triggered, whether fatigue alone is sufficient to increase self-reported episodic discontinuation intention remains an open question.

In this study, episodic discontinuation intention is treated not as a directly observed behavioral outcome, but as a self-reported psychological state that reflects users’ momentary intention to discontinue browsing during ongoing use, rather than a long-term or permanent withdrawal from the platform. The within-subject analyses show that emotional fatigue exerts a significant and robust positive effect on episodic discontinuation intention [*β* = 0.385, SE = 0.046, *t* = 8.41, CI (0.295, 0.475)]. This suggests that even in a short-term task context, fatigue is already meaningfully associated with users’ judgments about whether they want to keep browsing.

At the same time, episodic discontinuation intention should not be equated with permanent exit. It is better understood as a short-term, state-like orientation toward pausing or stepping away from the current stream of use. Fatigue, in this sense, does not indicate an actual discontinuation from the platform. Instead, it increases the user’s self-reported intention to discontinue browsing during ongoing use. Whether this tendency develops into actual discontinuation is still likely to depend on other conditions, including behavioral costs, task demands, and individual regulatory strategies.

Taken together, these findings suggest that fatigue is better understood not as an automatic trigger of discontinuance, but as an affective condition that increases the user’s self-reported intention to intermittently discontinue browsing. They also indicate that the path from discontinuation intention to actual behavior remains contingent, and therefore still requires further investigation.

### 6.4 The sequential psychological process

Overall, the central contribution of this study lies not in demonstrating whether fatigue leads to actual discontinuance, but in explicating how fatigue forms during use and under what conditions it becomes associated with discontinuation-related intention.

By situating differences in information quality within a continuous process involving cognitive load, use evaluation, and emotional experience, this study seeks to offer an explanatory pathway that is more closely aligned with users’ actual usage experiences.

## 7 Conclusion and implications

### 7.1 Conclusion

Instead of asking whether veracity or redundancy matters, this study asks when each cue matters and how its influence unfolds during browsing. Specifically, we examine the conditions under which, and the pathways through which, information quality cues shape user experience. The findings suggest that information quality cues do not immediately produce emotional reactions or discontinuation intention. Rather, their influence unfolds gradually: cognitive burden increases first, and only after this burden is interpreted at the evaluative stage does it acquire emotional meaning.

Importantly, cognitive load does not by itself lead to fatigue. Emotional fatigue emerges only when this burden is interpreted at the evaluative stage as a devalued usage experience, namely, when continued investment is perceived as no longer worthwhile. What ultimately shapes emotional outcomes is not the sheer intensity of processing, but how that experience is interpreted. In other words, fatigue is not a direct response to high processing intensity, but an affective reaction to a perceived decline in usage value.

### 7.2 Theoretical and practical implications

This study offers several theoretical implications. First, it advances research on information overload and social media discontinuance by shifting attention from external stressors and discontinuance outcomes to the psychological process through which information-quality cues shape users’ browsing experience. The findings suggest that information veracity and redundancy do not function merely as external triggers. Instead, they influence users’ cognitive load, evaluative interpretation, and emotional fatigue in a staged process.

Second, the study clarifies the role of negative use evaluation in this process. Negative use evaluation should not be equated with satisfaction or overall attitude. It reflects a situational judgment that continued investment has lost value during a specific browsing episode. This evaluative step helps explain how cognitive burden acquires emotional significance and develops into emotional fatigue.

Third, the findings suggest that the effects of information-quality cues may vary across psychological stages. At the evaluative stage, veracity and redundancy operate largely independently. At the emotional stage, however, high redundancy appears to reduce the difference in emotional fatigue between high- and low-veracity conditions. This stage-dependent pattern suggests that information cues are not integrated in the same way across the whole process.

From a practical perspective, the findings suggest that reducing users’ self-reported episodic discontinuation intention may require more than simply limiting information quantity. Social media platforms and content producers may need to improve information veracity, reduce repetitive content exposure, and help users more efficiently judge whether information is worth continued attention. In fragmented browsing environments, repeated exposure to uncertain and redundant content may gradually increase cognitive burden and emotional fatigue even during relatively short browsing episodes.

### 7.3 Limitations and directions for future research

Several limitations of this study warrant consideration and also suggest directions for future research.

First, the sample consisted exclusively of university students and graduate students aged 20–29. This relatively homogeneous sample constrains the direct generalizability of the findings to other age groups or populations with different educational backgrounds. Users from different demographic groups may systematically differ in information processing habits, allocation of cognitive resources, and tolerance for sustained social media use, which may alter how the psychological process identified here unfolds in practice. The choice of a student sample was motivated by their representativeness as heavy social media users and by the methodological advantage of controlling confounding factors at an initial model-validation stage. Future research should replicate and extend the proposed model in more diverse populations to assess its robustness across user groups. However, the present study did not systematically measure participants’ social media usage frequency, familiarity with travel-guide content, travel-planning involvement, or task motivation. Future research should include these variables to further examine how individual differences shape cognitive load, evaluation, and fatigue during browsing.

Second, the experimental materials in this study focused on travel-related information and were designed to simulate an information stream environment centered on experiential sharing and visual presentation. While this design enhanced experimental control and contextual consistency, the conclusions are currently grounded in this specific content domain and task structure. Future research should examine whether the proposed psychological process model holds across other information domains or usage contexts, thereby testing its stability under different task conditions.

Third, the experimental session lasted approximately 20 min, which was suitable for capturing participants’ immediate responses during a short browsing episode. However, this duration may not fully reflect longer-term social media use or the cumulative development of fatigue over repeated sessions. Future research should examine this process using longer tasks, longitudinal designs, or behavioral tracking data.

Finally, although the present study examined a cognitive–emotional process driven by information quality cues, it did not systematically account for individual differences in self-regulation strategies. Future research may examine how such differences shape the operation of the proposed process.

## Data Availability

The raw data supporting the conclusions of this article will be made available by the authors, without undue reservation.
