# Supplementary material for: From information quality to episodic discontinuation intention: cognitive and affective processes in social media browsing
Source: Front Psychol. 2026 Jul 2;17:1858297. doi: 10.3389/fpsyg.2026.1858297 (PMC13375218; doi:10.3389/fpsyg.2026.1858297)
Supplement: Supplementary file 1 [file Supplementary_file_1.docx]

**Appendix A**

**Table A1: Reliability and Validity Statistics of the Main Constructs**

| **Construct** | **Cronbach’s α** | **CR** | **AVE** | **√AVE** |
| --- | --- | --- | --- | --- |
| CL | .935 | .946 | .813 | .902 |
| NUE | .827 | .865 | .618 | .786 |
| EF | .886 | .921 | .745 | .863 |
| EDI | .777 | .867 | .686 | .828 |

Note: CR = composite reliability; AVE = average variance extracted. Standardized item loadings were close to or above .70. Reliability and validity statistics were calculated after removing CL5 and EDI1. Discriminant validity was supported using the Fornell-Larcker criterion.

**Table A2: Inter-Construct Correlations**

| **Construct** | **CL** | **NUE** | **EF** | **EDI** |
| --- | --- | --- | --- | --- |
| CL | 1.000 | .669 | .148 | .311 |
| NUE | .669 | 1.000 | .294 | .344 |
| EF | .148 | .294 | 1.000 | .355 |
| EDI | .311 | .344 | .355 | 1.000 |

**Table A3: Fornell-Larcker Matrix**

| **Construct** | **CL** | **NUE** | **EF** | **EDI** |
| --- | --- | --- | --- | --- |
| CL | **.902** | .669 | .148 | .311 |
| NUE | .669 | **.786** | .294 | .344 |
| EF | .148 | .294 | **.863** | .355 |
| EDI | .311 | .344 | .355 | **.828** |

**Appendix B: Experimental Materials and Manipulation Details**

| **Condition** | **Word Count** | **Images** | **Layout** | **Topic** | **Manipulation** |
| --- | --- | --- | --- | --- | --- |
| High veracity × Low redundancy | approx. 280 | same | same | same | verified concise text |
| High veracity × High redundancy | approx. 580 | same | same | same | repeated verified information |
| Low veracity × Low redundancy | approx. 280 | same | same | same | inaccurate/unverified fragments |
| Low veracity × High redundancy | approx. 580 | same | same | same | inaccurate/unverified fragments + repetitive elaboration |

**Appendix C: Revised Factor Loadings**

Promax-rotated Pattern Matrix^a^

|  | Component | | | |
| --- | --- | --- | --- | --- |
|  | 1 | 2 | 3 | 4 |
| CL2 | .928 |  |  |  |
| CL3 | .907 |  |  |  |
| CL1 | .889 |  |  |  |
| CL4 | .882 |  |  |  |
| EF3 |  | .881 |  |  |
| EF1 |  | .864 |  |  |
| EF2 |  | .862 |  |  |
| EF4 |  | .843 |  |  |
| NUE1 |  |  | .860 |  |
| NUE2 |  |  | .791 |  |
| NUE3 |  |  | .790 |  |
| NUE4 |  |  | .694 |  |
| EDI4 |  |  |  | .843 |
| EDI2 |  |  |  | .831 |
| EDI3 |  |  |  | .810 |

Extraction Method: Principal Component Analysis.

Rotation Method: Promax with Kaiser Normalization.

1. Rotation converged in 5 iterations.
