# Supplementary material for: From information quality to episodic discontinuation intention: cognitive and affective processes in social media browsing
Source: Front Psychol. 2026 Jul 2;17:1858297. doi: 10.3389/fpsyg.2026.1858297 (PMC13375218; doi:10.3389/fpsyg.2026.1858297)
Supplement: Supplementary file 2 [file Data_Sheet_1.PDF]

Supplementary File S1

Complete Experimental Materials

This file contains all experimental stimulus materials used in the study, including the main browsing page and all stimulus screenshots across the four experimental conditions.

Main page

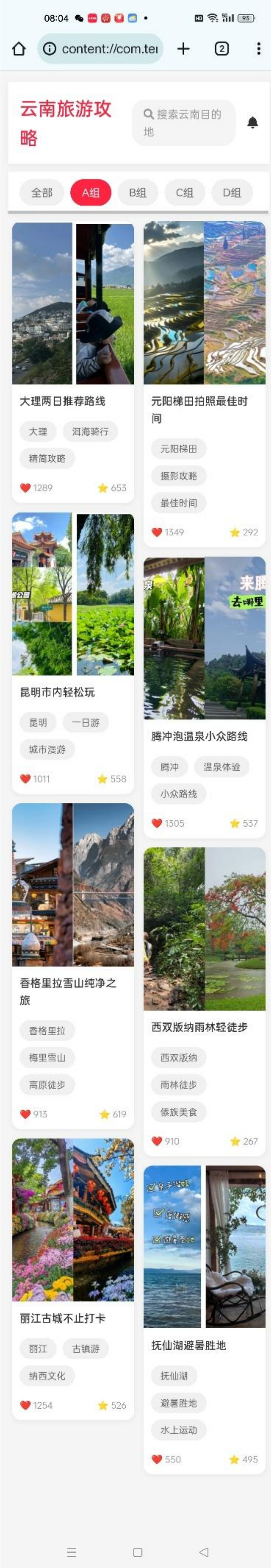
08:04
content://com.te
云南旅游攻略
搜索云南目的地
全部
A组
B组
C组
D组
大理两日推荐路线
大理
洱海骑行
精简攻略
1289
653
元阳梯田拍照最佳时间
元阳梯田
摄影攻略
最佳时间
1349
292
昆明市内轻松玩
昆明
一日游
城市漫游
1011
558
腾冲泡温泉小众路线
腾冲
温泉体验
小众路线
1305
537
香格里拉雪山纯净之旅
香格里拉
梅里雪山
高原徒步
913
619
西双版纳雨林轻徒步
西双版纳
雨林徒步
傣族美食
910
267
丽江古城不止打卡
丽江
古镇游
纳西文化
1254
526
抚仙湖避暑胜地
抚仙湖
避暑胜地
水上运动
550
495

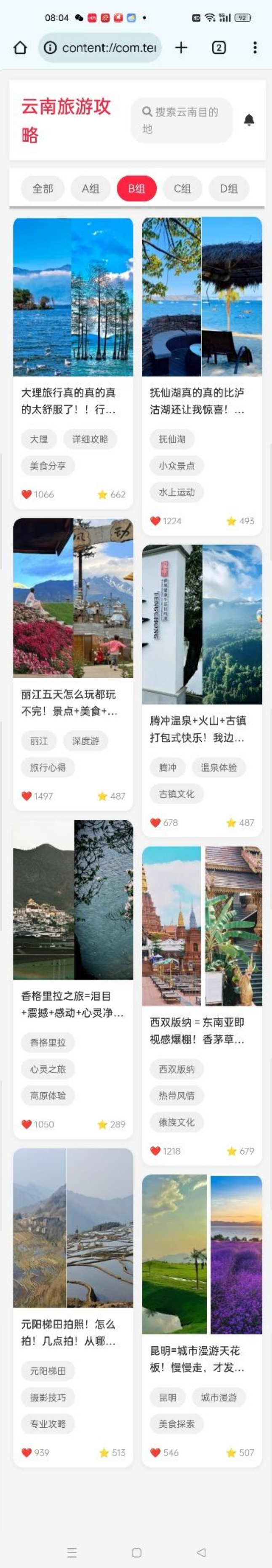
08:04
content://com.te
云南旅游攻略
搜索云南目的地
全部
A组
B组
C组
D组
大理旅行真的真的真的太舒服了！！行…
大理
详细攻略
美食分享
1066
662
抚仙湖真的真的比泸沽湖还让我惊喜！…
抚仙湖
小众景点
水上运动
1224
493
丽江五天怎么玩都玩不完！景点+美食+…
丽江
深度游
旅行心得
1497
487
腾冲温泉+火山+古镇打包式快乐！我边…
腾冲
温泉体验
古镇文化
678
487
香格里拉之旅=泪目+震撼+感动+心灵净…
香格里拉
心灵之旅
高原体验
1050
289
西双版纳 = 东南亚即视感爆棚！香茅草…
西双版纳
热带风情
傣族文化
1218
679
元阳梯田拍照！怎么拍！几点拍！从哪…
元阳梯田
摄影技巧
专业攻略
939
513
昆明=城市漫游天花板！慢慢走，才发…
昆明
城市漫游
美食探索
546
507

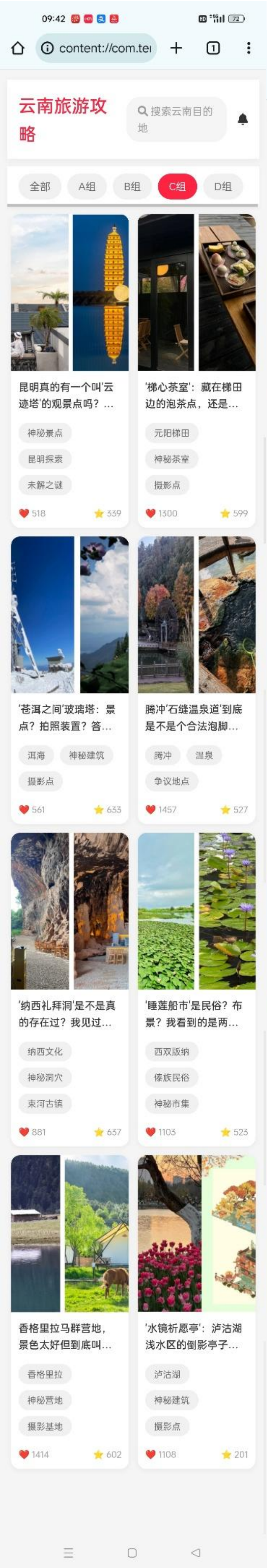
09:42
content://com.te
云南旅游攻略
搜索云南目的地
全部
A组
B组
C组
D组
昆明真的有一个叫'云迹塔'的观景点吗？…
神秘景点
昆明探索
未解之谜
518
339
'梯心茶室'：藏在梯田边的泡茶点，还是…
元阳梯田
神秘茶室
摄影点
1300
599
'苍洱之间'玻璃塔：景点？拍照装置？答…
洱海
神秘建筑
摄影点
561
633
腾冲'石缝温泉道'到底是不是个合法泡脚…
腾冲
温泉
争议地点
1457
527
'纳西礼拜洞'是不是真的存在过？我见过…
纳西文化
神秘洞穴
束河古镇
881
657
'睡莲船市'是民俗？布景？我看到的是两…
西双版纳
傣族民俗
神秘市集
1103
523
香格里拉马群营地，景色太好但到底叫…
香格里拉
神秘营地
摄影基地
1414
602
'水镜祈愿亭'：泸沽湖浅水区的倒影亭子…
泸沽湖
神秘建筑
摄影点
1108
201

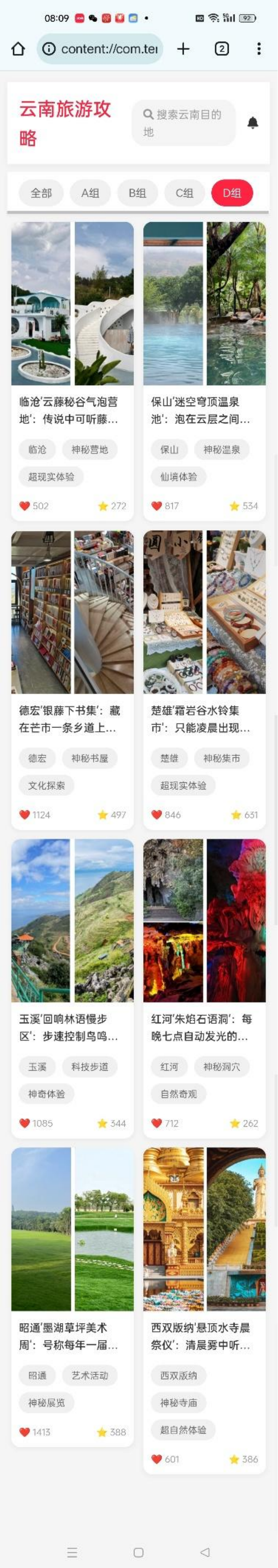
08:09
content://com.te
云南旅游攻略
搜索云南目的地
全部
A组
B组
C组
D组
临沧'云藤秘谷气泡营地'：传说中可听藤…
临沧
神秘营地
超现实体验
502
272
保山'迷空穹顶温泉池'：泡在云层之间…
保山
神秘温泉
仙境体验
817
534
德宏'银藤下书集'：藏在芒市一条乡道上…
德宏
神秘书屋
文化探索
1124
497
楚雄'霜岩谷水铃集市'：只能凌晨出现…
楚雄
神秘集市
超现实体验
846
651
玉溪'回响林语慢步区'：步速控制鸟鸣…
玉溪
科技步道
神奇体验
1085
344
红河'朱焰石语洞'：每晚七点自动发光的…
红河
神秘洞穴
自然奇观
712
262
昭通'墨湖草坪美术周'：号称每年一届…
昭通
艺术活动
神秘展览
1413
388
西双版纳'悬顶水寺晨祭仪'：清晨雾中听…
西双版纳
神秘寺庙
超自然体验
601
386

Condition A: High Veracity × Low Redundancy

A1-A8

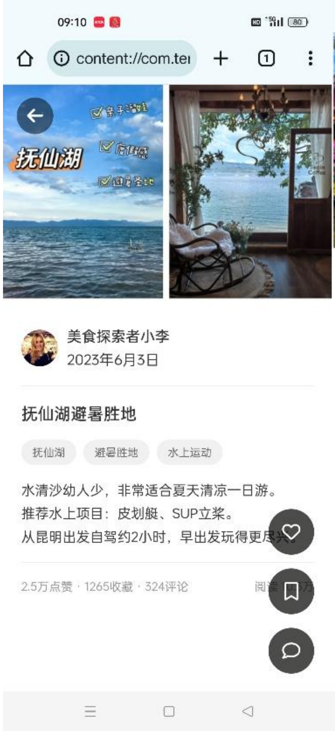

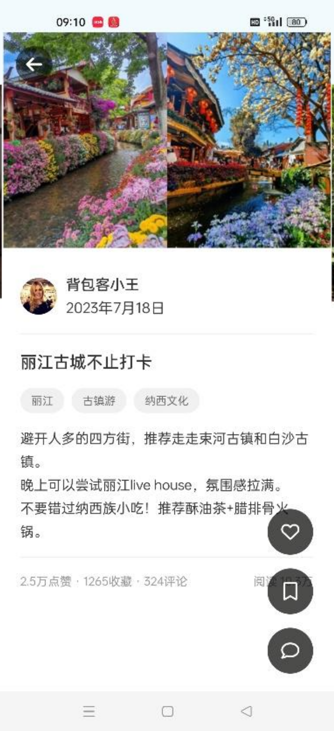

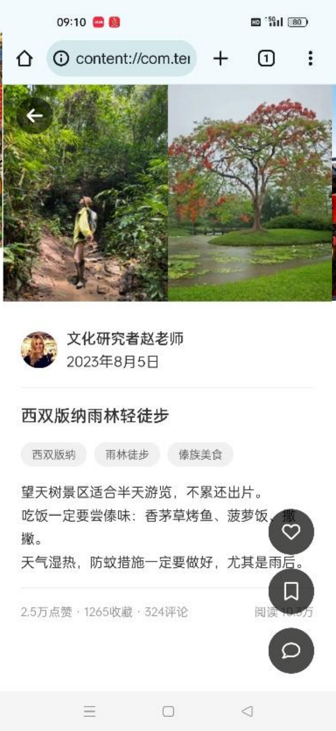

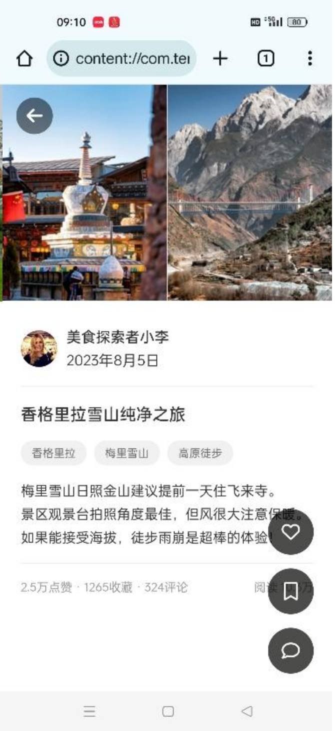

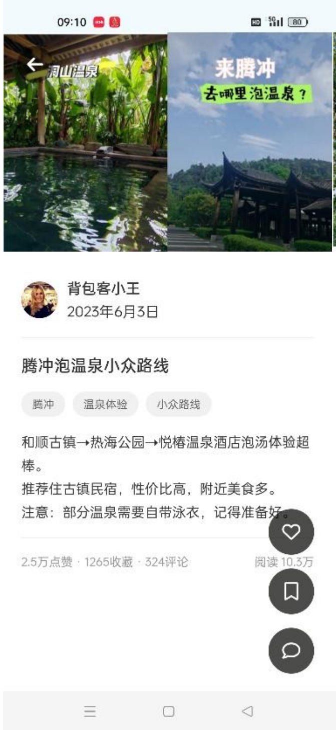

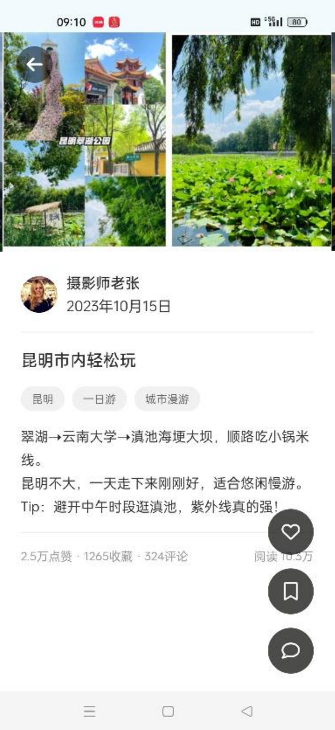

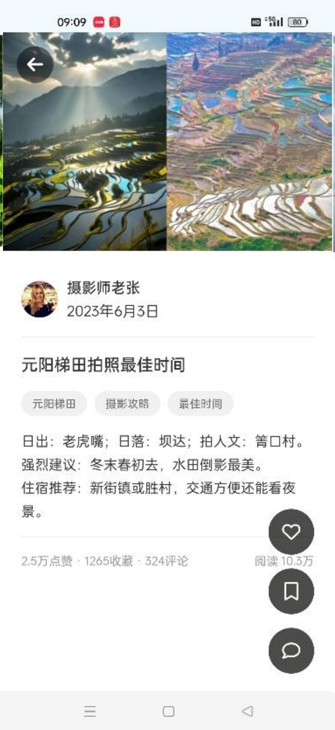

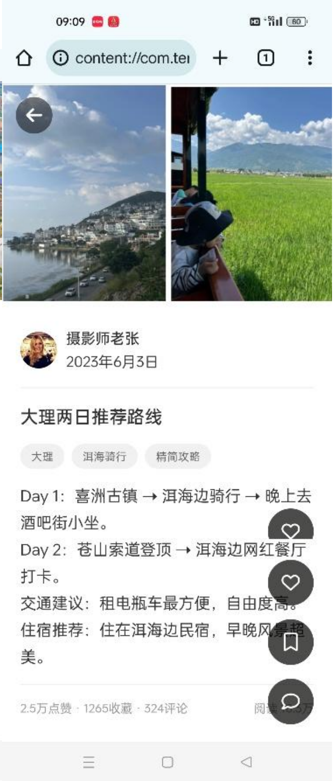

Condition B: High Veracity × High Redundancy

B1-B8

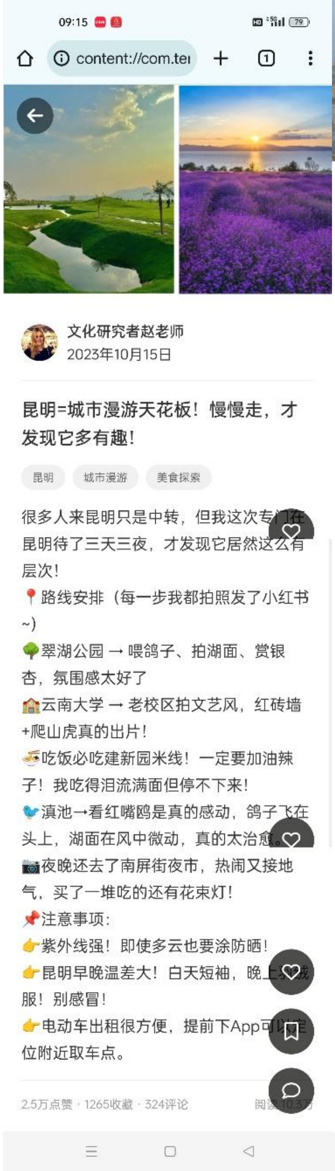

09:15

content://com.tei

文化研究者赵老师
2023年10月15日

昆明=城市漫游天花板！慢慢走，才发现它多有趣！

昆明 城市漫游 美食探索

很多人来昆明只是中转，但我这次专门在昆明待了三天三夜，才发现它居然这么有层次！
📍路线安排（每一步我都拍照发了小红书~）
🌳翠湖公园 → 喂鸽子、拍湖面、赏银杏，氛围感太好了
🏫云南大学 → 老校区拍文艺风，红砖墙+爬山虎真的出片！
🍜吃饭必吃建新园米线！一定要加油辣子！我吃得泪流满面但停不下来！
🐦滇池→看红嘴鸥是真的感动，鸽子飞在头上，湖面在风中微动，真的太治愈[illegible]
📷夜晚还去了南屏街夜市，热闹又接地气，买了一堆吃的还有花束灯！
📌注意事项：
👉紫外线强！即使多云也要涂防晒！
👉昆明早晚温差大！白天短袖，晚上[illegible]服！别感冒！
👉电动车出租很方便，提前下App可以[illegible]位附近取车点。

2.5万点赞 · 1265收藏 · 324评论 阅读[illegible]

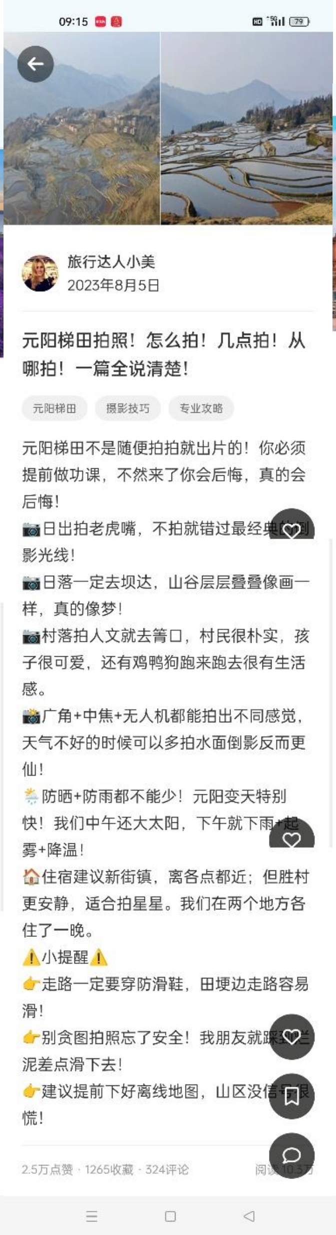

09:15

旅行达人小美
2023年8月5日

元阳梯田拍照！怎么拍！几点拍！从哪拍！一篇全说清楚！

元阳梯田 摄影技巧 专业攻略

元阳梯田不是随便拍拍就出片的！你必须提前做功课，不然来了你会后悔，真的会后悔！
📷日出拍老虎嘴，不拍就错过最经典[illegible]影光线！
📷日落一定去坝达，山谷层层叠叠像画一样，真的像梦！
📷村落拍人文就去箐口，村民很朴实，孩子很可爱，还有鸡鸭狗跑来跑去很有生活感。
📸广角+中焦+无人机都能拍出不同感觉，天气不好的时候可以多拍水面倒影反而更仙！
🌦防晒+防雨都不能少！元阳变天特别快！我们中午还大太阳，下午就下雨+起雾+降温！
🏠住宿建议新街镇，离各点都近；但胜村更安静，适合拍星星。我们在两个地方各住了一晚。
⚠小提醒⚠
👉走路一定要穿防滑鞋，田埂边走路容易滑！
👉别贪图拍照忘了安全！我朋友就踩[illegible]泥差点滑下去！
👉建议提前下好离线地图，山区没信[illegible]慌！

2.5万点赞 · 1265收藏 · 324评论 阅读[illegible]

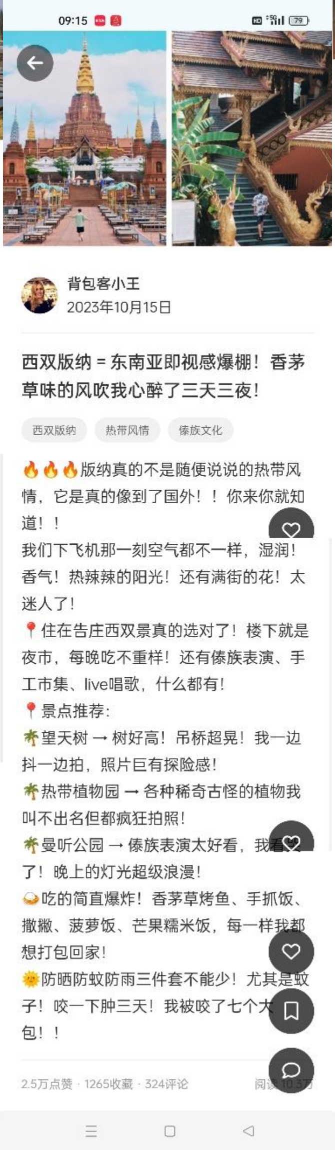

09:15

背包客小王
2023年10月15日

西双版纳 = 东南亚即视感爆棚！香茅草味的风吹我心醉了三天三夜！

西双版纳 热带风情 傣族文化

🔥🔥🔥版纳真的不是随便说说的热带风情，它是真的像到了国外！！你来你就知道！！
我们下飞机那一刻空气都不一样，湿润！香气！热辣辣的阳光！还有满街的花！太迷人了！
📍住在告庄西双景真的选对了！楼下就是夜市，每晚吃不重样！还有傣族表演、手工市集、live唱歌，什么都有！
📍景点推荐：
🌴望天树 → 树好高！吊桥超晃！我一边抖一边拍，照片巨有探险感！
🌴热带植物园 → 各种稀奇古怪的植物我叫不出名但都疯狂拍照！
🌴曼听公园 → 傣族表演太好看，我[illegible]了！晚上的灯光超级浪漫！
🍛吃的简直爆炸！香茅草烤鱼、手抓饭、撒撇、菠萝饭、芒果糯米饭，每一样我都想打包回家！
🌞防晒防蚊防雨三件套不能少！尤其是蚊子！咬一下肿三天！我被咬了七个大包！！

2.5万点赞 · 1265收藏 · 324评论 阅读[illegible]

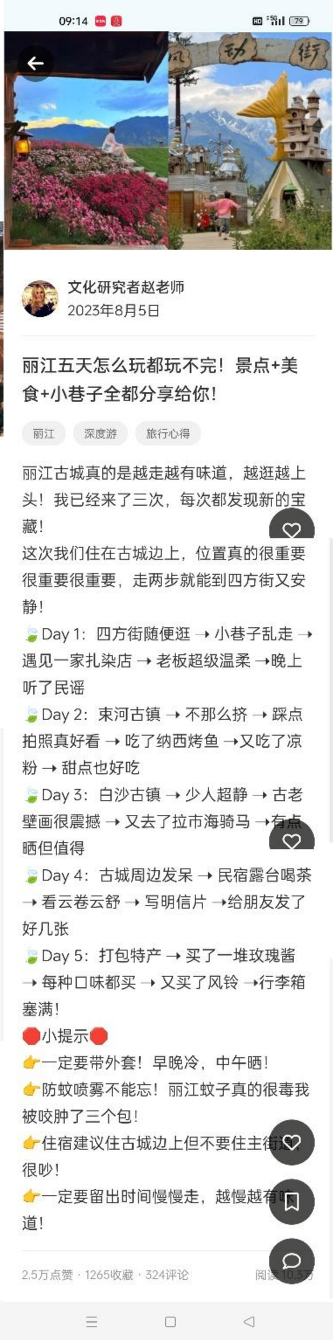

09:14

文化研究者赵老师
2023年8月5日

丽江五天怎么玩都玩不完！景点+美食+小巷子全都分享给你！

丽江 深度游 旅行心得

丽江古城真的是越走越有味道，越逛越上头！我已经来了三次，每次都发现新的宝藏！
这次我们住在古城边上，位置真的很重要很重要很重要，走两步就能到四方街又安静！
🍃Day 1：四方街随便逛 → 小巷子乱走 → 遇见一家扎染店 → 老板超级温柔 →晚上听了民谣
🍃Day 2：束河古镇 → 不那么挤 → 踩点拍照真好看 → 吃了纳西烤鱼 →又吃了凉粉 → 甜点也好吃
🍃Day 3：白沙古镇 → 少人超静 → 古老壁画很震撼 → 又去了拉市海骑马 →有点晒但值得
🍃Day 4：古城周边发呆 → 民宿露台喝茶 → 看云卷云舒 → 写明信片 →给朋友发了好几张
🍃Day 5：打包特产 → 买了一堆玫瑰酱 → 每种口味都买 → 又买了风铃 →行李箱塞满！
🔴小提示🔴
👉一定要带外套！早晚冷，中午晒！
👉防蚊喷雾不能忘！丽江蚊子真的很毒我被咬肿了三个包！
👉住宿建议住古城边上但不要住主街[illegible]很吵！
👉一定要留出时间慢慢走，越慢越有[illegible]道！

2.5万点赞 · 1265收藏 · 324评论 阅读[illegible]

09:14

content://com.te

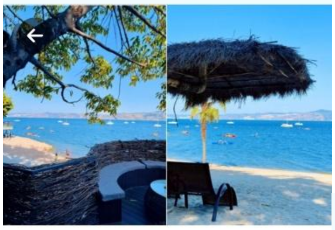

背包客小王
2023年10月15日

抚仙湖真的真的比泸沽湖还让我惊喜！水清！天蓝！人少！我狂拍400张图！

抚仙湖 小众景点 水上运动

姐妹们听我一句劝！抚仙湖！！太小众！太干净！太舒服！太适合夏天了！！真的比我预期高太多！！
我们早上从昆明出发🚗，自驾两个小时就到了，一路蓝天白云+绿树成荫，真的像动画片背景一样。
到了之后，我们第一句话就是'哇也太清了吧！'水清得像镜子，真的能看见脚趾头那种！！
🌊项目安排如下（划重点！！！）
✔️SUP立式划桨 → 超好拍！我站在板上旋转了10圈，朋友拍了200张！
✔️皮划艇 → 湖面超静，真的像在童话世界！
✔️环湖骑行 → 太惬意了，每骑5分钟就想停下来拍照！
✔️沙滩野餐 → 拍照、晒太阳、吹风、发呆，全部安排上！
🌅建议住一晚！湖边小民宿真的太香了！落地窗+阳台+蓝天，晚上听水声入眠！早上睁眼就是湖光！
📷拍照tips：白色长裙yyds、草帽一定要戴、大耳环真的点睛、光脚站水里真[illegible]围感爆棚！！
📢注意事项📢
👉强烈建议早到早玩！中午开始紫外线爆表！
👉吃的推荐铜锅鱼+小炒牛肉+豆花[illegible]线，搭配当地酸梅汁，超清爽！
👉水上项目怕晒的朋友要穿防晒衣，[illegible]黑两度但值得！

2.5万点赞 · 1265收藏 · 324评论 阅[illegible]

09:14

content://com.te

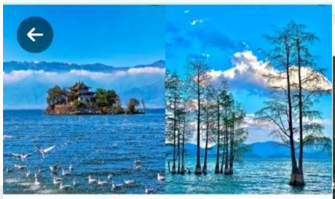

旅行达人小美
2023年8月5日

大理旅行真的真的真的太舒服了！！行程+美食+拍照全分享！

大理 详细攻略 美食分享

姐妹们我说真的！大理是我这几年去过最最放松、最治愈的地方！真的超适合散心、放空、拍照、发呆、骑车、赏花、喝咖啡、吹风发呆发呆再发呆！
我们这次的行程如下👇（非常推荐，真的不要错过！）：
📍Day 1：大理古城 → 四方街随便逛逛 → 洱海边拍拍拍 → 晚上去酒吧街坐坐 → 回民宿阳台聊天喝茶
📍Day 2：喜洲古镇 → 蓝花楹大道 → 拍白墙蓝天 → 吃白族三道茶 → 喝饵丝 → 买明信片 → 晚上洗衣服
📍Day 3：苍山索道 → 坐到山顶真的冷 → 拍雪景 → 下山又吃了一次过桥米[illegible]
‼️‼️‼️重点来了‼️‼️‼️
☀️防晒！必须防晒！物理+化学+衣物三重叠加！我晒爆皮！！
📸穿浅色系更上镜，白裙子超级适合洱海背景，蓝天白云yyds！！
⛱️建议租电瓶车骑洱海环海西路，记[illegible]前电量充满，真的超大一圈！
🍲饵丝真的好吃，早中晚都能吃，而[illegible]同店味道都不一样，你们试试！

2.5万点赞 · 1265收藏 · 324评论 [illegible]

09:36

content://com.te

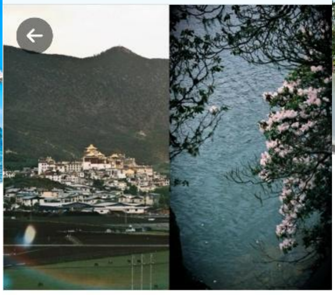

背包客小王
2023年8月5日

香格里拉之旅=泪目+震撼+感动+心灵净化的一次旅行

香格里拉 心灵之旅 高原体验

我真的不知道怎么形容香格里拉这个地方，太纯净、太神圣、太宁静、太震撼了，真的一秒心灵洗涤那种感觉！！
我们在飞来寺住了两晚就为了看一次梅里雪山日照金山，结果！真的看到了！我哭了！
🗻第一天：飞来寺看星星 → 第二天五点起来拍日照金山 → 冻成狗但太值得！
🛕后来去松赞林寺 → 沿路拍照每一张都像明信片 → 气氛庄重 → 虔诚震撼
🍲吃饭也感动：酥油茶、牦牛火锅、青稞饼我都爱，每一口都暖心又暖胃！
🚶最后我们徒步了雨崩 → 真的是原始森林 → 每走一步都是震撼 → 每喘一口气都觉得神圣
🌙晚上我们在山里看星星 → 满天星[illegible]银河真的看得见 → 人生第一次
📸拍照拍爆 → 自拍→拍雪山→拍转经[illegible]→拍落日→拍影子→拍牦牛→全拍！

2.5万点赞 · 1265收藏 · 324评论 [illegible]

09:35

content://com.te

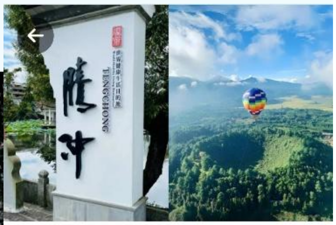

美食探索者小李
2023年10月15日

腾冲温泉+火山+古镇打包式快乐！我边泡边感叹：也太舒服了吧？！

腾冲 温泉体验 古镇文化

如果你问我云南哪最养生、最放松、最[illegible]得冬天去，那我一定回答你：腾冲！
我们是11月底去的，一下飞机就闻到了硫磺味（别怕，很快就习惯了），说明温泉是真的。
📍第一站：和顺古镇。古镇是真的古，巷子又小又安静，随便走走都像穿越。我们看了书店、听了戏、买了火山石肥皂，太有意思了。
📍第二站：悦椿温泉。OMG这个温泉我必须吹爆！有露天、有花瓣池、有牛奶池、有蒸汽屋、有足疗池、还有泡脚石路，[illegible]从下午泡到天黑！！泡到发！困！泡到不想动！
📍第三站：热海公园+火山公园！喷泉真的喷得老高了！像开锅一样咕噜咕噜响，配火山地貌，真的震撼！
📍第四站：夜市小吃街，一口气吃了大救驾、腾冲饵丝、凉拌猪脚、豆粉、酸汤牛肉，我真是边走边吃后悔为什么只有一个胃！
🧖泡温泉建议：提前预约！带拖鞋毛巾泳衣！手机带防水袋！泡完记得喝水多休息！

2.5万点赞 · 1265收藏 · 324评论 [illegible]

Condition C: Low Veracity × Low Redundancy

C1-C8

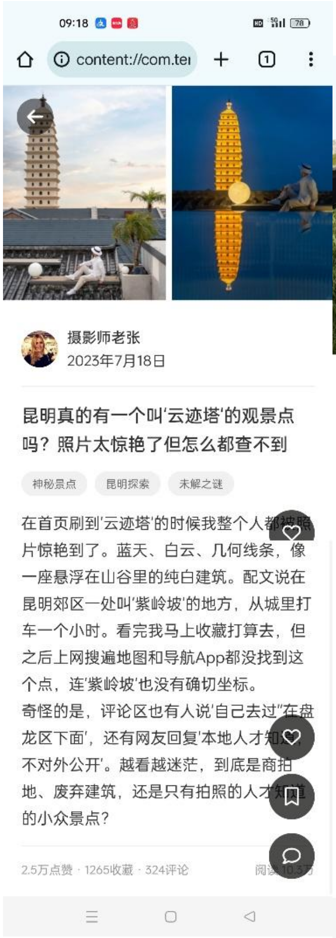

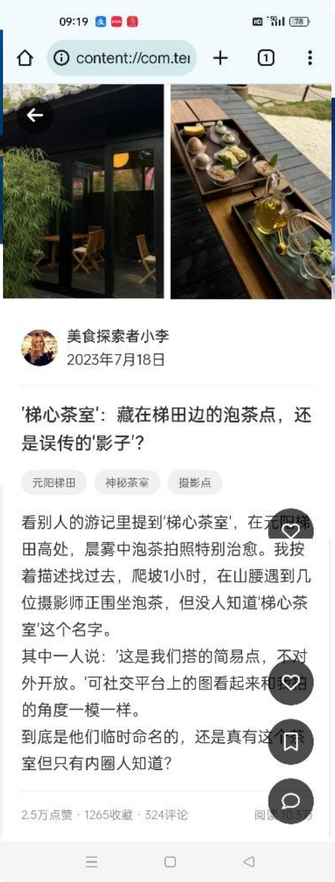

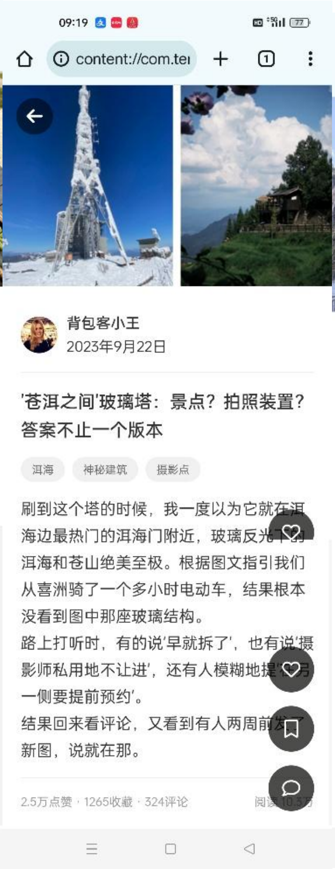

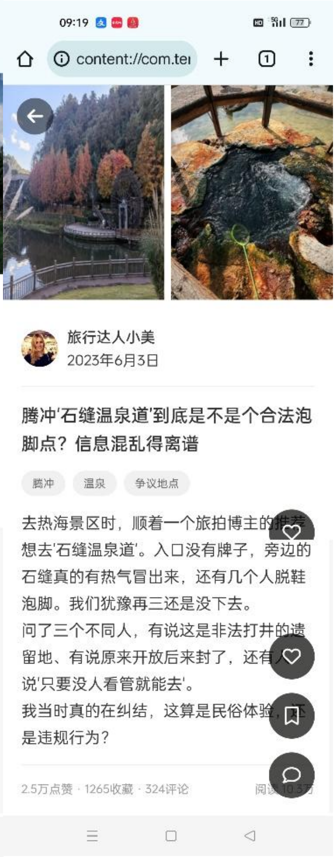

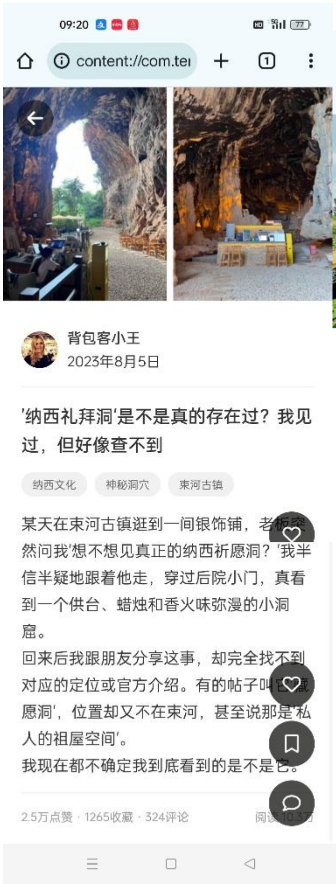

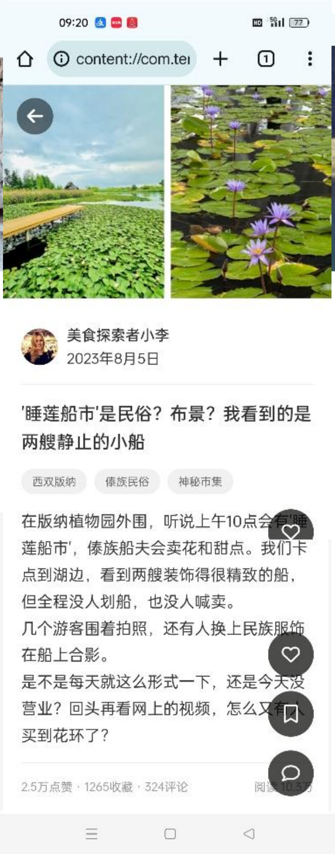

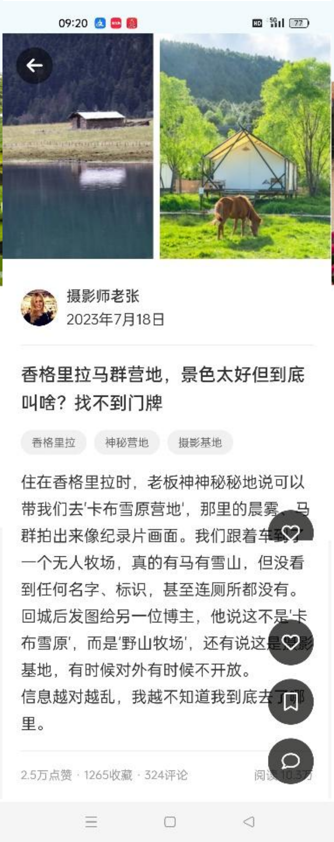

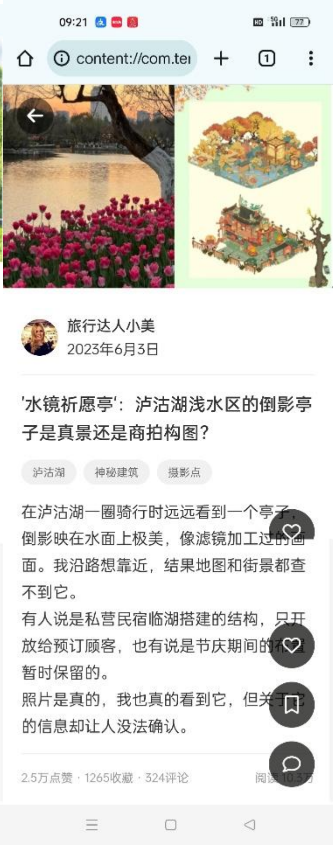

Condition D: Low Veracity × High Redundancy

D1-D8

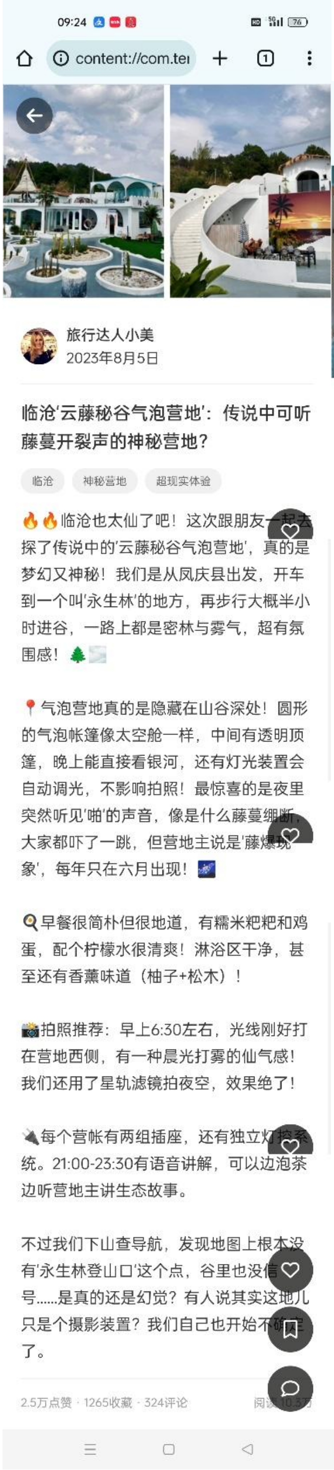

09:24

content://com.te

旅行达人小美
2023年8月5日

临沧'云藤秘谷气泡营地'：传说中可听藤蔓开裂声的神秘营地？

临沧 神秘营地 超现实体验

🔥🔥临沧也太仙了吧！这次跟朋友一起去探了传说中的'云藤秘谷气泡营地'，真的是梦幻又神秘！我们是从凤庆县出发，开车到一个叫'永生林'的地方，再步行大概半小时进谷，一路上都是密林与雾气，超有氛围感！🌲🌫️

📍气泡营地真的是隐藏在山谷深处！圆形的气泡帐篷像太空舱一样，中间有透明顶篷，晚上能直接看银河，还有灯光装置会自动调光，不影响拍照！最惊喜的是夜里突然听见'啪'的声音，像是什么藤蔓绷断，大家都吓了一跳，但营地主说是'藤爆现象'，每年只在六月出现！🌌

🍳早餐很简朴但很地道，有糯米粑粑和鸡蛋，配个柠檬水很清爽！淋浴区干净，甚至还有香薰味道（柚子+松木）！

📸拍照推荐：早上6:30左右，光线刚好打在营地西侧，有一种晨光打雾的仙气感！我们还用了星轨滤镜拍夜空，效果绝了！

🔌每个营帐有两组插座，还有独立灯控系统。21:00-23:30有语音讲解，可以边泡茶边听营地主讲生态故事。

不过我们下山查导航，发现地图上根本没有'永生林登山口'这个点，谷里也没信号……是真的还是幻觉？有人说其实这地儿只是个摄影装置？我们自己也开始不[illegible]了。

2.5万点赞 · 1265收藏 · 324评论 阅读10.3万

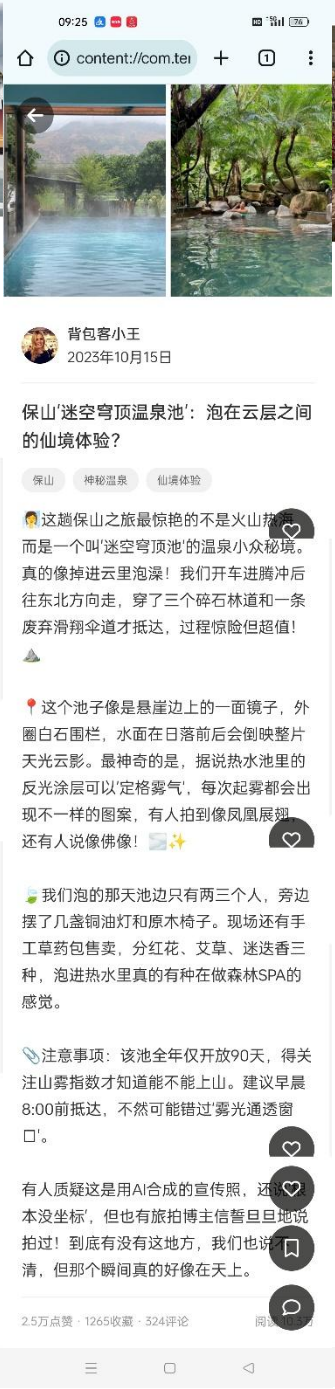

09:25

content://com.te

背包客小王
2023年10月15日

保山'迷空穹顶温泉池'：泡在云层之间的仙境体验？

保山 神秘温泉 仙境体验

🧖这趟保山之旅最惊艳的不是火山热海，而是一个叫'迷空穹顶池'的温泉小众秘境。真的像掉进云里泡澡！我们开车进腾冲后往东北方向走，穿了三个碎石林道和一条废弃滑翔伞道才抵达，过程惊险但超值！⛰️

📍这个池子像是悬崖边上的一面镜子，外圈白石围栏，水面在日落前后会倒映整片天光云影。最神奇的是，据说热水池里的反光涂层可以'定格雾气'，每次起雾都会出现不一样的图案，有人拍到像凤凰展翅，还有人说像佛像！🌫️✨

🍃我们泡的那天池边只有两三个人，旁边摆了几盏铜油灯和原木椅子。现场还有手工草药包售卖，分红花、艾草、迷迭香三种，泡进热水里真的有种在做森林SPA的感觉。

📎注意事项：该池全年仅开放90天，得关注山雾指数才知道能不能上山。建议早晨8:00前抵达，不然可能错过'雾光通透窗口'。

有人质疑这是用AI合成的宣传照，还说'根本没坐标'，但也有旅拍博主信誓旦旦地说拍过！到底有没有这地方，我们也说不清，但那个瞬间真的好像在天上。

2.5万点赞 · 1265收藏 · 324评论 阅读10.3万

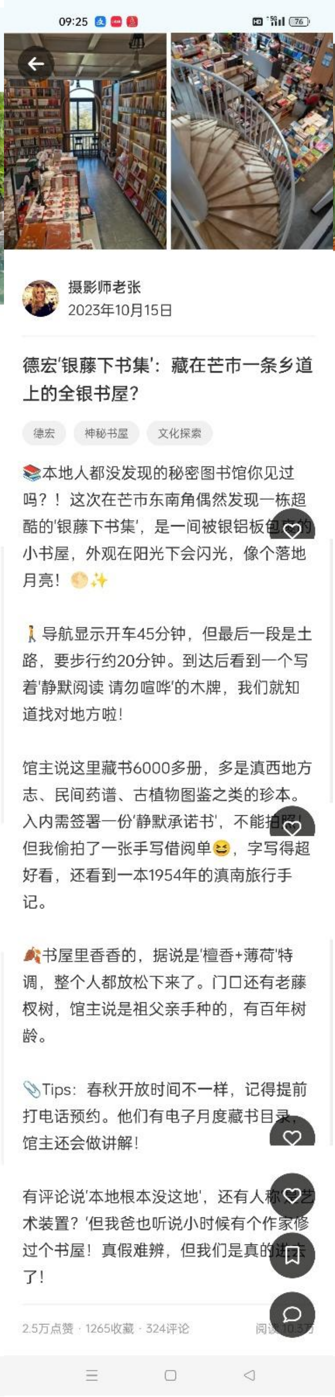

09:25

摄影师老张
2023年10月15日

德宏'银藤下书集'：藏在芒市一条乡道上的全银书屋？

德宏 神秘书屋 文化探索

📚本地人都没发现的秘密图书馆你见过吗？！这次在芒市东南角偶然发现一栋超酷的'银藤下书集'，是一间被银铝板包[illegible]的小书屋，外观在阳光下会闪光，像个落地月亮！🌕✨

🚶导航显示开车45分钟，但最后一段是土路，要步行约20分钟。到达后看到一个写着'静默阅读 请勿喧哗'的木牌，我们就知道找对地方啦！

馆主说这里藏书6000多册，多是滇西地方志、民间药谱、古植物图鉴之类的珍本。入内需签署一份'静默承诺书'，不能拍照！但我偷拍了一张手写借阅单😆，字写得超好看，还看到一本1954年的滇南旅行手记。

🍂书屋里香香的，据说是'檀香+薄荷'特调，整个人都放松下来了。门口还有老藤杈树，馆主说是祖父亲手种的，有百年树龄。

📎Tips：春秋开放时间不一样，记得提前打电话预约。他们有电子月度藏书目录，馆主还会做讲解！

有评论说'本地根本没这地'，还有人称[illegible]艺术装置？'但我爸也听说小时候有个作家修过个书屋！真假难辨，但我们是真的[illegible]了！

2.5万点赞 · 1265收藏 · 324评论 阅读10.3万

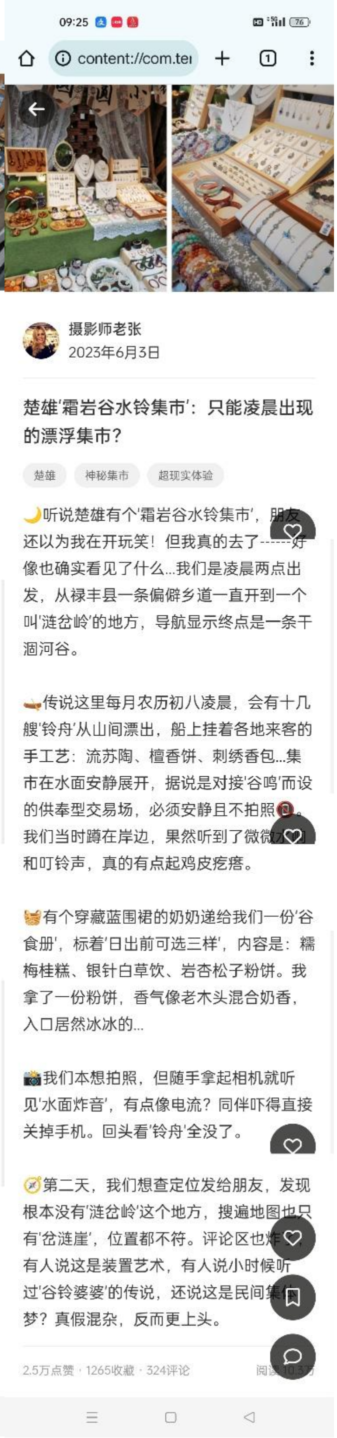

09:25

content://com.te

摄影师老张
2023年6月3日

楚雄'霜岩谷水铃集市'：只能凌晨出现的漂浮集市？

楚雄 神秘集市 超现实体验

🌙听说楚雄有个'霜岩谷水铃集市'，朋友还以为我在开玩笑！但我真的去了------好像也确实看见了什么...我们是凌晨两点出发，从禄丰县一条偏僻乡道一直开到一个叫'涟岔岭'的地方，导航显示终点是一条干涸河谷。

🛶传说这里每月农历初八凌晨，会有十几艘'铃舟'从山间漂出，船上挂着各地来客的手工艺：流苏陶、檀香饼、刺绣香包...集市在水面安静展开，据说是对接'谷鸣'而设的供奉型交易场，必须安静且不拍照📵。我们当时蹲在岸边，果然听到了微微[illegible]和叮铃声，真的有点起鸡皮疙瘩。

🧺有个穿藏蓝围裙的奶奶递给我们一份'谷食册'，标着'日出前可选三样'，内容是：糯梅桂糕、银针白草饮、岩杏松子粉饼。我拿了一份粉饼，香气像老木头混合奶香，入口居然冰冰的...

📸我们本想拍照，但随手拿起相机就听见'水面炸音'，有点像电流？同伴吓得直接关掉手机。回头看'铃舟'全没了。

🧭第二天，我们想查定位发给朋友，发现根本没有'涟岔岭'这个地方，搜遍地图也只有'岔涟崖'，位置都不符。评论区也炸了，有人说这是装置艺术，有人说小时候听过'谷铃婆婆'的传说，还说这是民间集[illegible]梦？真假混杂，反而更上头。

2.5万点赞 · 1265收藏 · 324评论 阅读10.3万

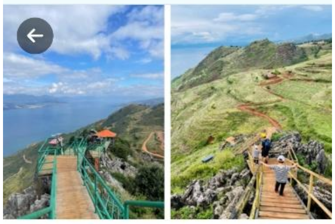

文化研究者赵老师
2023年8月5日

玉溪'回响林语慢步区'：步速控制鸟鸣的魔法步道？

玉溪 科技步道 神奇体验

🌿玉溪真的很会搞！这次走了一条叫'回响林语'的步道，超级神奇，它居然能根据你走路的快慢自动播放不同鸟鸣声？！！！

📍入口在'月桂西坪'停车点，从那里起步，步道两侧挂着小装置，说是声音捕捉器和反射器，我们一开始还以为是监控，结果走快了真的听见'唧------'一声，好像是鹰鹃？？慢下来就变成'啾啾啾'，像竹鸡！！太酷了！🦜

🎧据说是结合AI和生态感应技术，步[illegible]配套了同步APP，能显示你当前的步速和推荐音频。我们下载后，还能记录你走过的'鸣区'，像打卡一样好玩！

⚠️有碑文提示'请勿用手电筒直射'，我们不懂就照了一下，结果音频暂停了！吓死我了！😨

🧢建议穿防水登山鞋、探光帽，步速保持在1.5km/h，最容易触发'森林深语'模式，感觉置身电影场景！

很多人留言说'听说过但不确定在哪里'，也有说'去了没听见鸟'，甚至有人觉得'鸟鸣是预录的？'但我们是真的听到了那种随[illegible]切换的声音。

2.5万点赞 · 1265收藏 · 324评论 阅[illegible]

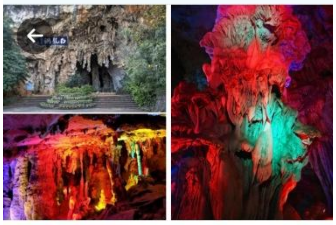

摄影师老张
2023年6月3日

红河'朱焰石语洞'：每晚七点自动发光的彩岩秘境？

红河 神秘洞穴 自然奇观

✨在红河元阳县，有一个叫'朱焰石语洞'的地方，真的被我无意间发现！原本是去多依树看梯田，结果在半路拐到一条没铺柏油的支路上，路边贴着手写小牌子：'往朱焰石洞 1.4km'。好奇心驱使我们走进了一片石林，居然走到一个像被火烧过的山洞口！

🌋这个洞不深，三四米就到底，但墙体全是红褐色石纹，夹杂着星星点点的银亮颗粒。我们刚进洞什么都没看见，但刚好过七点，整个洞壁像打了灯一样发出淡红光，像岩浆涌动，还有一种若隐若现的'嗡嗡'声。带耳机的朋友说听起来像是老磁带倒带的声音...👀

💡我当时还以为是幻觉，结果翻出网上帖子，有人说这叫'矿岩光感异相'，红河部分地段岩层含微量电气石成分，潮湿后遇气温骤降就能短时间'释放电磁辉光'⚡。真假无从考证，但现场的感觉，真的太震撼！

📍实用信息：洞口在'垭垭坪村'后山[illegible]道，导航无法准确定位，需在'盘阳梯田观景台'左侧岔口步行入林。建议携带微光手电+强制线地图app。建议17:30前进洞，保持安静。

📸附：最佳拍照时机是19:03--19:15之间，此时光线最稳定。使用延时快门+三脚架可以拍出类似岩浆流动的特效。我们还试着拍了光轨，结果闪出类似'蝌蚪符号'图样？！

🗣️传闻：'朱焰'之名来自早年间一个[illegible]的采矿队员梦游至此失踪，后来又在别处突然现身，说'洞会说话'。这个故事在村里老一辈中流传至今。

⛔但最离谱的是，我们第二天再找，那个手写牌子居然没了！有人评论说这只是[illegible]光团队搞的'视觉干预项目'，也有人说这洞早就塌了。到底是哪里来的幻觉？我[illegible]始怀疑人生...

2.5万点赞 · 1265收藏 · 324评论 阅[illegible]

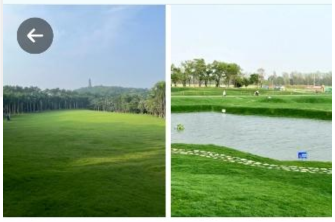

文化研究者赵老师
2023年9月22日

昭通'墨湖草坪美术周'：号称每年一届却查无具体主办？

昭通 艺术活动 神秘展览

🎨刚从昭通回来，竟然误打误撞参与了所谓的'第五届墨湖草坪美术周'？一开始是看到路边指示牌写着'墨草2025'，抱着好奇心跟着走进去，结果发现一片草地上真有很多装置艺术！帐篷画展、投影秀、还发了入场编号和纸质地图！？

现场有一堵'历届主题墙'，还有速写比赛区、草地演讲亭，夜幕降临后还有灯光装置和手绘涂鸦投影，看着很像是正规文艺节，参与者也都很专业的样子。

⛺他们还有草地露营区、自由画板区、[illegible]作交换站等，我甚至看到有人手上戴着所谓'历年收藏手环'，上面写着'墨草2023'。

📎展区布图、活动流程表、风力棚搭建图纸，全都印得特别正式，还有个小棚子专门展示以前活动的'官方纪念票根'。

但我回来一查，网上根本没有'墨湖'的具体位置，也查不到任何政府或机构主办信息。评论区也众说纷纭，有人说'朋友[illegible]过'，也有人说'像一群艺术生自办的'，但这么大场面，真能只是临时搭的吗？越[illegible]玄......

2.5万点赞 · 1265收藏 · 324评论 阅[illegible]

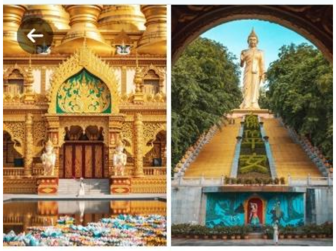

文化研究者赵老师
2023年6月3日

西双版纳'悬顶水寺晨祭仪'：清晨雾中听梵音的幻觉还是真实？

西双版纳 神秘寺庙 超自然体验

🛕谁能想到，在热闹的西双版纳热带雨林里，还藏着一座'悬顶水寺'？这次我和朋友从景洪出发，沿着一条老旧的乡道走了将近两个小时，来到传说中的'笼南寨外山麓'，找这座'只在清晨显现'的水寺遗址。

🚶我们凌晨4:30出发，5:45左右抵达山脚，周围浓雾弥漫，能见度不到5米。正当我们怀疑是不是被骗时，耳边传来一种低频梵唱------像极了寺庙钟声与流水交织。我们顺着声音走，迷雾中果然看到一处池水映出三角屋顶样的影子，水波微动，影子像在'升起'一样。✨

🌫️同行有个学地理的朋友说，这地方地形特殊，加上日出湿度变化，会出现'悬顶反光幻觉'，但也有人说那影子是真的'水上庙宇'，平时浸没于池底，只在特定天气与角度下显现。真假难辨，但我们全程录音中确实采集到规律性诵经节奏！

🗨️现场还有几块布满老傣文的石碑，一位穿红衣的老人突然出现，自称'照守人'，[illegible]给我们一份叫'晨祭仪'的小册子，上面详细写着早祭时间表、手势顺序、念诵节拍，甚至连跪拜方向、所穿衣色（推荐月白或赭红）都有说明！

📸附录内容：最佳拍摄时间为6:10--6:30，推荐使用滤光偏振镜拍摄水面可见'虚实交叠'；温度需在22℃以下，湿度80%以上才容易出现完整反影。

🔍但是我们离开后上网查，完全找不到'笼南寨'这个村庄，也没有'悬顶水寺'的任何官方或民间资料。有网友留言称这是近年流行的'声场错觉旅拍地'，靠隐形音响与雾化玻璃制造错觉，还有人说这个'照守人'是一个流浪艺术家扮的，早在微博上被人扒过。

但亲眼所见的那一刻，真的感觉自己置身某种界限之外。回想起来，甚至怀疑[illegible]个旅程的记忆都被'雾'改写了......

2.5万点赞 · 1265收藏 · 324评论 阅[illegible]
